# Effectiveness of a Brief Internet-Based Workplace Training Program to Support Help Seeking for Mental Ill-Health: Cluster Randomized Controlled Trial

**DOI:** 10.2196/89215

**Published:** 2026-08-07

**Authors:** Philip J Batterham, Amelia Gulliver, Cassandra Heffernan, Alison L Calear, Aliza Werner-Seidler, Alyna Turner, Louise M Farrer, Mary Lou Chatterton, Cathrine Mihalopoulos, Monica Gendi, Michael Berk

**Affiliations:** 1 Centre for Mental Health Research, The Australian National University, Canberra, Australian Capital Territory, 2600, Australia, 61 26125 ext 1031; 2 Black Dog Institute, University of New South Wales, Sydney, New South Wales, Australia; 3 Deakin Institute for Mental and Physical Health and Clinical Translation (IMPACT), School of Medicine, Faculty of Health, Deakin University, Geelong, Victoria, Australia; 4 School of Public Health and Preventive Medicine, Monash University, Melbourne, Victoria, Australia; 5 Orygen, The National Centre of Excellence in Youth Mental Health, Centre for Youth Mental Health, Florey Institute for Neuroscience and Mental Health and the Department of Psychiatry, The University of Melbourne, Melbourne, Victoria, Australia

**Keywords:** help seeking, mental health, workplace, employee, implementation, internet, psychiatry, psychology

## Abstract

**Background:**

Depression and anxiety are prevalent in working-aged adults. Although treatment provided by health professionals can improve symptoms and functioning, many people experiencing mental ill-health do not seek help. There have been very few effective interventions to improve help seeking in adults, with none implemented across diverse workplaces through online delivery.

**Objective:**

The primary aim of this trial was to test the effectiveness of a co-designed program for increasing professional help seeking intentions in Australian employees, relative to an active control condition.

**Methods:**

A triple-blinded, 2-arm cluster randomized controlled trial (N=487; control workplaces: n=26 and intervention workplaces: n=25) was conducted to assess the relative effectiveness of Helipad, a fully automated co-designed single-session interactive program (intervention condition) with a standard psychoeducation program (active control condition). Eligible workplaces (those with ≥50 employees and an employee assistance program; clusters) were recruited via advertising or invited directly by researchers. Eligible participants (employees in a participating Australian workplace, aged ≥18 y, living in Australia, fluent in reading and understanding English, and had access to a device and a reliable internet connection) completed a pretest, immediate posttest, and 6-month follow-up survey sent via email assessing help seeking intentions (primary outcome); mental illness stigma; mental health literacy; help seeking attitudes and behavior; work and activity functioning; quality of life; and symptoms of depression, anxiety, and general psychological distress.

**Results:**

A significant difference in change over time in professional help seeking intentions was found between the 2 conditions (*F*_2,185.44_=6.89; *P*=.001), with planned contrasts showing that the Helipad program was effective in increasing professional help seeking intentions compared with the control at the primary end point of immediate posttest (*t*_359.35_=−3.72; *P*<.001). This difference was not maintained at the 6-month follow-up (*t*_119.76_=−1.05; *P*=.30). Retention rates were 71.1% at posttest and 24.9% at follow-up. The Helipad program was also associated with improved mental health literacy and help seeking attitudes at posttest. Helipad was not significantly superior to the control in reducing mental illness stigma or improving professional help seeking behavior; functioning; quality of life; or symptoms of depression, anxiety, or general psychological distress (secondary outcomes) at the 6-month follow-up. A total of 15 people (control: n=9, 4.5%; intervention: n=6, 4.3%) reported adverse events at posttest.

**Conclusions:**

This study demonstrated that the co-designed online Helipad program was effective in improving the intentions of employees to seek help from a professional compared to an active control. The program also improved mental health literacy and help seeking attitudes, but these changes were not sustained and did not translate into observable differences in help seeking behaviors or mental health symptoms. This was the first trial of a co-designed intervention to improve mental health help seeking in general workplaces, with previous trials focusing primarily on specific high-risk work environments. Workplace programs that can be delivered at scale may lead to more appropriate engagement with professional mental health care to prevent poor mental health outcomes and increase productivity.

## Introduction

### Background

Mental disorders remain in the top 10 leading causes of burden worldwide, with recent data showing a prevalence of 12% of people experiencing a mental disorder globally [1]. Nearly half of Australian adults will experience a mental disorder during their lifetime; 21.5% during any 12-month period [2]. Anxiety disorders (17.2%) and major depressive episodes (4.9%) are the most commonly experienced mental disorders in a single year [2]. Worldwide, working-aged adults experience the greatest rates of mental disorders, with 80.6% of the burden of disease from mental disorders occurring in working-aged adults (16‐65 y) [1]. In Australia, the prevalence of mental disorders is highest in young adults aged 16 to 24 years (38.8%) and 25 to 34 years (26.3%), with those aged 55 to 64 years experiencing a lower, although still high, 17.9% prevalence of mental disorders in a year [2].

Despite the high rates of all mental disorders, fewer than half of Australian adults experiencing symptoms seek help from a health professional [2]. Typically, even when people do seek help, many have experienced symptoms over a long period before seeing a health professional [3,4], with delays in treatment reported to be 6 to 8 years for depression and 9 to 23 years for anxiety disorders. A lack of appropriate professional support may affect the person’s ability to recover, resulting in poorer outcomes for mental health, lower quality of life, increased issues within social and family networks, and potentially issues at work such as functional impairment and productivity loss [5-9]. Effective treatment also has benefits for general health. For example, recent data suggest that in those with depressive symptoms, use of antidepressants was associated with an increase in disability-free survival of 2.95 years (95% CI 2.12‐3.04) [10].

These gaps in service use are driven by a range of barriers [11]. Some are impacted by structural factors such as cost, poor service accessibility, waiting lists, transport, childcare [11], or digital poverty [12]. Sociocultural barriers include the experience of generational trauma, poor past help seeking experiences, historical medical mistreatment, or a lack of culturally appropriate, trauma-informed services, and masculine gender norms [13-15]. However, there are also personal and attitudinal factors that can impact a person’s likelihood to seek help when needed, including stigmatizing attitudes toward mental ill-health and help seeking [16], self-stigma [17], a lack of knowledge about help seeking and mental health in general, termed “mental health literacy,” and a preference to manage mental health symptoms on their own [18,19]. Attitudinal barriers are thought to be particularly problematic for help seeking in mild-to-moderate mental health issues [11]. People who have experienced early childhood trauma or neglect are also more likely to have disrupted attachments and consequent difficulties with trust and help seeking [20].

The global costs of untreated depression and anxiety disorders are projected to be more than 12 billion days of lost productivity and more than US $1 trillion per year [21]. In Australia, estimated economic costs of mental ill-health, which is made up of workplace absenteeism, presenteeism (ie, attending work while ill), and compensation claims, are estimated to be Aus $11 billion (US $7.3 billion) per year [22]. Of the working adult population, Australian data from 2021 to 2022 indicated that 9% of all serious workers’ compensation claims and 7% of all work-related injuries and illnesses were from mental health conditions, a 36.9% increase since 2017 to 2018 [23]. Recent Australian legislation supports laws to prevent harm in the workplace from psychosocial hazards [24]. Common mental health problems can follow a chronic, relapsing, and treatment-resistant course, particularly if not treated early [25]. Thus, workplace mental health education programs to promote help seeking are critical.

Universal mental health promotion programs are a form of prevention that are delivered to all people in a particular group regardless of symptom levels [26]. Owing to this, they have the potential to significantly improve mental health by providing resources for those who are currently experiencing symptoms and equipping those who may experience such problems in the future [27]. Universal help seeking programs usually include information that targets specific modifiable barriers to seeking help. These include mental health literacy, including how, where, and when to seek help, and materials designed to improve stigmatizing attitudes toward mental illness and help seeking [27,28]. Accordingly, the World Health Organization recommends the provision of universal training for organizations, managers, and employees to improve mental health–related knowledge, improve stigmatizing attitudes in workplaces, and encourage help seeking [29].

To date, there have been few help seeking programs evaluated in employees broadly; many previous programs have been designed to target specific occupation groups, such as health care workers, firefighters, or construction workers [30-33]. Despite significant benefits of digital programs, including cost-effectiveness [34] and ability to be broadly disseminated [35], there have been only 2 online programs that have been evaluated in general workplace settings [36], neither having been implemented post evaluation. There remains a lack of evidence for the effectiveness of programs to improve help seeking more broadly across workplaces [31] and a lack of research on factors that support the implementation of such programs. Co-design is now recognized as an important method to bring significant expertise into the process of creating services [37]. Partnering with end users throughout program design and development may lead to more effective services [38] and potentially provide important information to facilitate improved research translation and implementation [37].

### Aims

This study tests the effectiveness of a novel co-designed program for improving help seeking in workplaces using a cluster randomized controlled trial.

The primary aim of the study was to test the effectiveness of a co-designed mental health help seeking program (the “Helipad” program) on increasing professional help seeking intentions (primary outcome) in employees in a workplace setting immediately after completing the program at posttest (primary end point) and a 6-month follow-up.

The secondary aims were to determine whether the program reduces mental illness stigma and improves help seeking attitudes and mental health literacy at posttest and the 6-month follow-up and improves professional help seeking behavior, mental health symptoms, quality of life, and functioning (secondary outcomes) relative to the control condition at the 6-month follow-up. A further exploratory aim was to examine moderators of help seeking intentions.

## Methods

### Trial Design

A 2-arm cluster randomized controlled trial (RCT) was used to assess the effectiveness of Helipad compared with standard psychoeducation alone on help seeking intentions by employees in workplaces. A protocol has been published describing the methodology of the trial [39], and the trial was prospectively registered on March 13, 2023 (Australian New Zealand Clinical Trials Registry ACTRN12623000270617 [40]), and participants were enrolled from May 6, 2024, to February 3, 2025.

### Ethical Considerations

The ethical aspects of this research were reviewed and approved by the Australian National University Human Research Ethics Committee (ANU HREC protocol 2023/053).

Checklist 1 presents the CONSORT (Consolidated Standards of Reporting Trials) EHEALTH checklist for reporting randomized web-based trials [41], and Checklist 2 presents the CONSORT checklist. Checklist 3 presents a completed intervention description and replication (TIDieR [Template for Intervention Description and Replication]) checklist [42]. Potential participants were provided with an information and consent page online, where they completed written consent before commencing the research. Survey data were stored on a secure server, as participants’ email addresses were collected for follow-up purposes but later deidentified. We did not provide incentives or payments for participation to either workplaces or individual participants. No identification of individual participants was possible in any images or other material in the manuscript or supplementary material.

### Interventions

#### The Helipad Program

We actively involved people with lived experience of mental ill-health in the co-design of the program, as well as those with other forms of experience in workplace settings (ie, management, human resources [HR], and mental health clinicians). A working group was assembled in 2023 and was led by lived experience researchers (AG and CH), who cofacilitated and led the co-design working group using iterative participatory methods to develop the content and form of the intervention. A separate publication describes this process in detail [43]. Briefly, we used a community-based co-design approach to guide the creation of the program with 9 co-designers (6 multiple roles of consumers, 5 carers, 2 managers, 2 human resources staff, and 1 clinician) who were currently employed [44]. Workshops comprised five 90-minute online workshops between May and October 2023, with the final 2 workshops including members of the external web development team to assist the group in identifying functional and feasible tools for the program that were within budget.

The co-designed program named “Helipad” adopted the funded project name of “Help seeking enhancement through Lived experience Participatory Design.” The Helipad program was a fully automated program that was undertaken individually and took approximately 20 minutes to complete. It was specifically designed to be brief to reduce barriers to completion in busy workplace schedules. The program included interactive quizzes and visual tools, printable lists, videos, and links to external web pages such as government-provided information on workplace and general guides on mental health, how to find a general practitioner (GP) or psychologist in Australia, and online mental health treatment programs. The co-design process identified 5 core issues that were important to encourage help seeking in workplace settings, which formed the 5 key modules of the program, described in detail previously [39,43] and outlined below.

**Figure 1.**
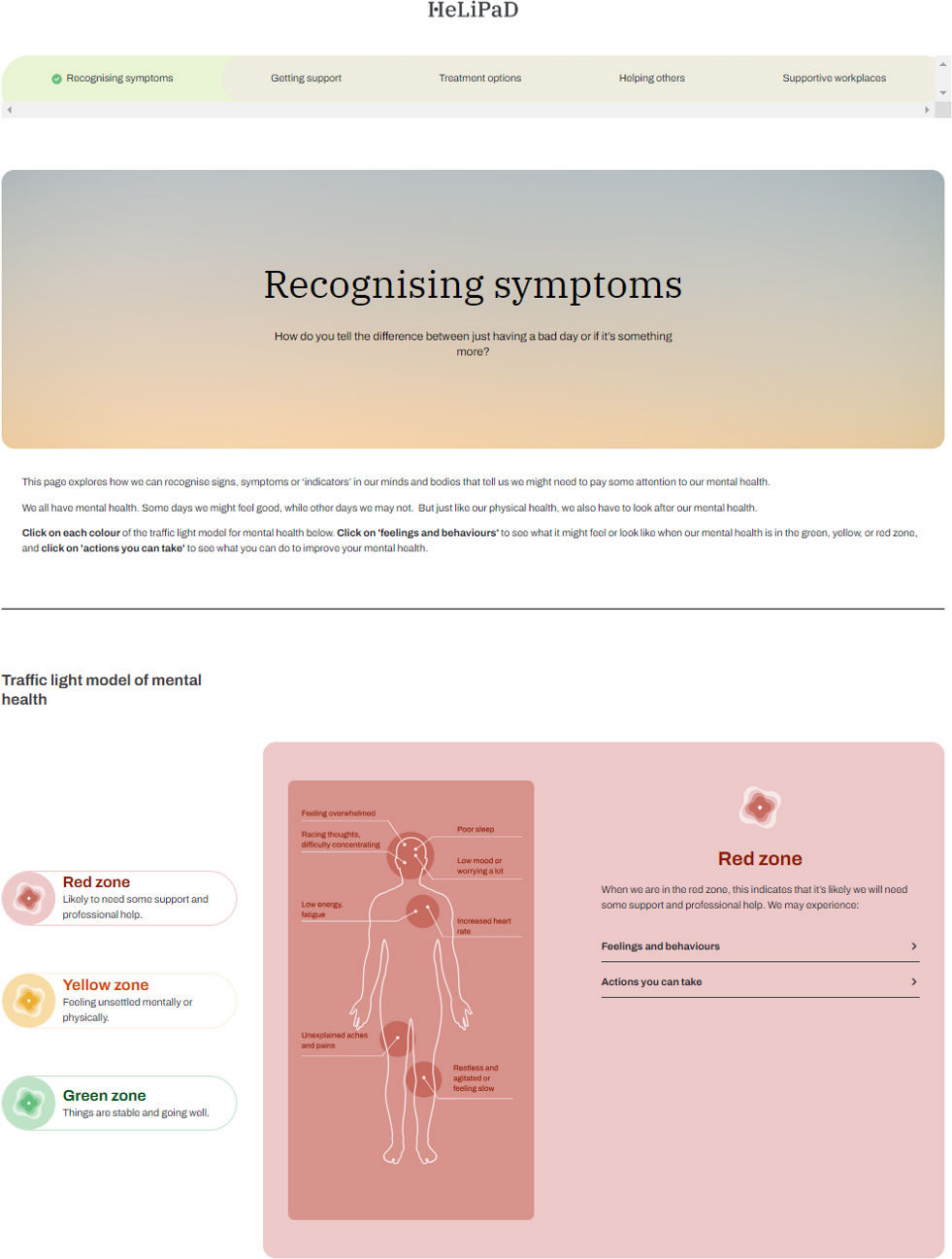
Screenshot from Helipad program “Recognizing symptoms“ (module 1) demonstrating the interactive “traffic light” model.

1. Recognizing symptoms: The first module used an interactive “traffic light” model to help employees recognize symptoms of different phases of mental health. It highlighted parts of the body and mind where we might identify signs, symptoms, or “indicators,” ranging from green (stable and well) to yellow (feeling unsettled) to red (likely needs support or professional help). Figure 1 displays a screenshot of module 1.
2. Getting support: Module 2 provided information to “demystify” the process of seeking help, including written information on how employees can access support and what support is offered by different types of professionals (GP, psychologist, employee assistance program [EAP], allied health professionals [eg, occupational therapist and social worker], peer worker, counselor, and psychiatrist). This included videos (EAP, psychologist, and GP) and a specific example pathway of an Australia-wide service model on how to seek help from a psychologist with or without a referral from a GP.
3. Treatment options: Module 3 comprised written information on evidence-based treatments (psychological, medication, and exercise and lifestyle) for common mental health problems (eg, anxiety and depression). A diagram of how to enact “cognitive restructuring” from CBT was included, along with a brief interactive exercise to identify people in employees’ lives who could support them in challenging times.
4. Helping others: Module 4 provided suggestions for how employees can help others in the workplace who disclose experiences of mental ill-health. It also included scenarios, including the impact of potentially helpful and unhelpful responses from colleagues and managers. A short video featured a manager who shared how they supported an employee who disclosed a mental health issue.
5. Supportive workplaces: The final module targeted stigma and concerns about workplace disclosure. It included 3 genuine videos of lived experience peers describing what happened when they disclosed a mental health issue at work, an interactive quiz debunking myths around depression and anxiety disorders, and guidance to alleviate concerns such as being uncomfortable talking about mental health.

#### Active Control Program

An active control condition was used to provide a robust test of efficacy for the co-designed, interactive elements of the Helipad program over and above the effect of text-based psychoeducation. We also expected that using a control condition that provided mental health information would increase both organizational interest in the trial and individual participant engagement. The researchers created a time-matched 5-module control program consisting of standard written psychoeducation on mental health and well-being. As in previous RCTs [45], the written information in the control program was primarily derived from the National Institutes of Health (NIH) News in Health newsletters [46] and minimally edited by the research team; however, for this trial, mental health content was deliberately included to create an active control comparator.

**Figure 2.**
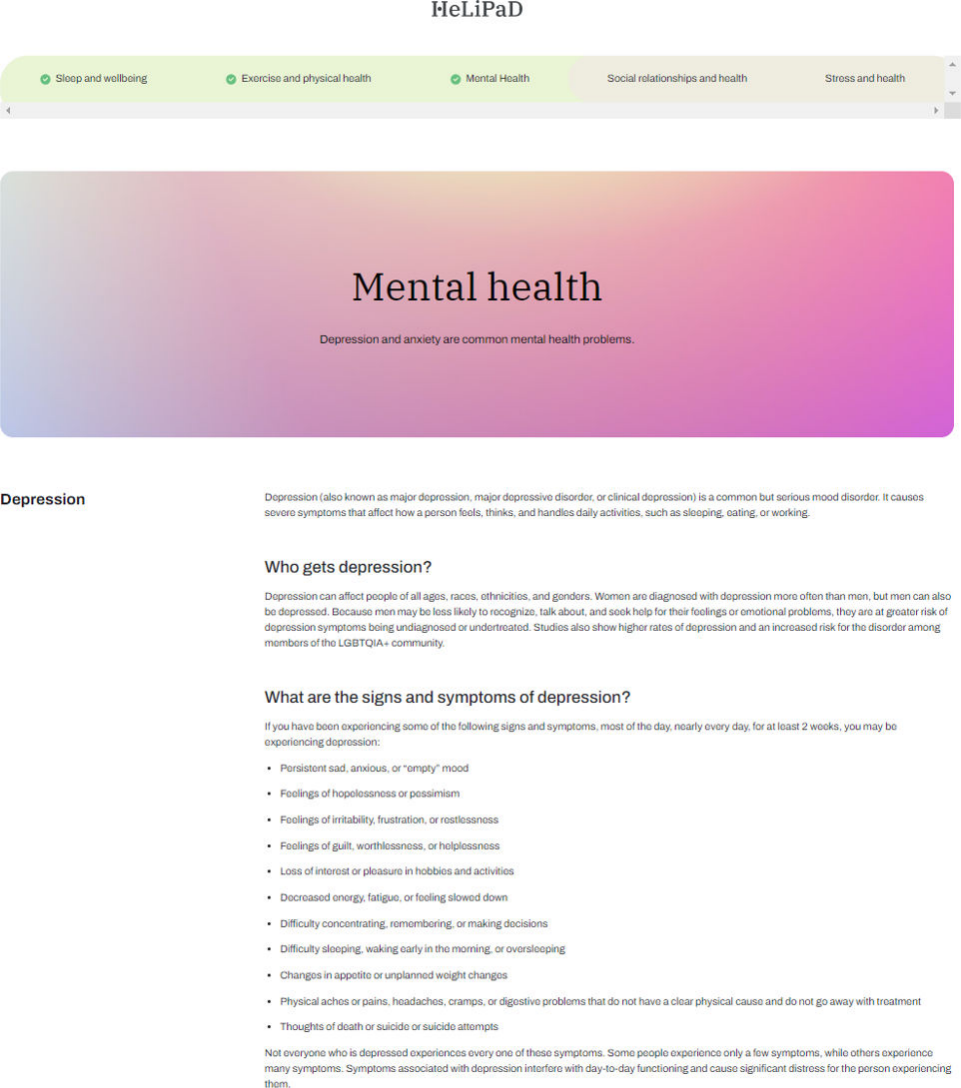
Screenshot from the control program of “Mental health” (module 3).

Of note, the control program was not co-designed, did not include detailed information about potential treatments for depression or anxiety, how or where to seek support from health professionals, or content explicitly targeting stigma reduction (eg, contact with people with lived experience via peer story sharing [47]). We expected that an active control condition providing psychoeducation would be likely to increase mental health literacy and potentially contribute to the reduction of stigma, but given previous research, we did not expect this to markedly impact help seeking intentions [48], providing a robust test for the primary outcome. The 5 modules of the control program included written psychoeducation about:

1. Sleep and well-being: This module provided information targeting the relationship of sleep to the health of our mind and body. It also included specific information on sleep stages, sleep quality and duration, and how these can impact overall health and well-being.
2. Exercise and physical health: This module included information about physical activity and exercise and how it can improve our health and well-being. This module also included motivational tips and strategies for increasing movement and overall physical activity.
3. Mental health: This module contained specific information about depression and anxiety disorders. It provided information on how to recognize if you might be experiencing depression or an anxiety disorder by including physical, behavioral, and mental signs and symptoms and briefly outlining potential treatment approaches. Figure 2 shows a screenshot of this module.
4. Social relationships and health: This module comprised information about how social relationships impact our health both positively and negatively, including how social connections buffer against stress, reporting on a scientific study where social connections appeared to reduce the development of cold symptoms after exposure to a virus.
5. Stress and health: The final module focused on providing information on the relationship between stress and health. It included common sources of stress and how people might reduce stress in daily life, such as increasing exercise, sleep, improving diet, and social contacts. It also specified mindfulness as a strategy to relieve stress.

### Procedure

#### Trial Setting

The trial was conducted online, with participants recruited via workplaces (ie, cluster RCT). The trial was delivered using 2 custom-designed websites that contained the self-report assessments, the 2 online programs, and email reminders, to be delivered seamlessly to participants. The websites also collected and securely housed the survey data on an online platform that the researchers could use to download data. The web development team who created the trial delivery platform used a separate paid service called “PostMark” to manage the automatic delivery of email reminders for the trial. Researchers could also send manual email reminders directly from the trial platform.

To streamline access and reduce the need for participants to create a personal account, while maintaining security and confidentiality, participants only needed to provide an email to access the program. If surveys or the program were discontinued, participants were emailed a single reminder (at 48 h if they discontinued any part of the pretest survey, program, and posttest survey; 1 wk for the 6-mo follow-up survey) to continue where they left off to complete that part of the study, with a unique link to access the program from their email account. Participants who completed the posttest surveys typically completed the pretest survey, program, and posttest survey in one sitting.

#### Participants

A total of 487 (control: n=264 and intervention: n=223) participants were recruited via 51 randomized eligible workplaces (control: n=26 and intervention: n=25) between May 2024 and February 2025. The first participant was enrolled on May 6, 2024.

#### Workplace Recruitment

Recruitment of workplaces was conducted using multiple methods, including direct approaches and online advertising.

##### Direct Approaches

We used multiple recruitment methods. Initially, we contacted existing networks via email and sent invitations through the Mental Health Australia General Clinical Trials (MAGNET) network. Next, we compiled a spreadsheet of 657 individual workplaces sourced from internet searches across a range of 16 broad industry types (eg, training and education, computing and IT, and property and construction). Typically, we first conducted a telephone call to identify appropriate contacts (eg, manager or HR lead), followed by a formal letter (via email) from the lead researcher and a workplace consent form. Later, we developed a trial information package comprising screenshots of the programs, a trial flow figure, frequently asked questions, and clear explanations of what was involved for both workplaces and employees. We also conducted searches of staff members in HR management roles on Sales Navigator and LinkedIn, then sent direct invitations to these individuals.

##### Advertising

We also used paid and unpaid broad social media advertising on LinkedIn and targeted paid invitation messages. We targeted advertisements to specific workplace roles (ie, HR managers and chief executive officers) and used the LinkedIn direct message function to send direct invitations to people in specific workplace roles (eg, HRs). We also shared the advertisement via our own personal LinkedIn pages and from the MAGNET trial platform page.

##### Registration Form

Requesting workplace representatives to send an email to register interest was not a highly successful strategy. Instead, a link to a brief Qualtrics registration form was included in paid advertisements and direct messages, which collected the individual’s name (optional), workplace name (optional), and email (compulsory), providing a streamlined method for registering interest. We then sent out tailored invitation letters and the trial information pack to these contacts.

Workplace representatives expressing interest through any of the abovementioned methods were contacted by the researchers via telephone or email to identify organization characteristics, assess eligibility for the study, and negotiate timing for the commencement of the trial.

### Workplace Eligibility

Workplaces were eligible if they (1) reported having at least 50 employees to ensure that each cluster was viable and (2) had an EAP available for their staff to ensure that employees experiencing mental health concerns had a clear pathway to care. Workplaces also needed to provide a signed copy of a “workplace agreement form,” which confirmed their understanding of all relevant aspects of the trial, and that their workplace met the inclusion criteria. We also collected data on workplace type (office based vs non–office based), number of employees (<200 vs ≥200 employees), location (combining New South Wales, Victoria, and the Australian Capital Territory vs other state or territory), and whether the workplace was primarily metropolitan or regionally based. Any industry of work was eligible for inclusion.

### Randomization

Workplaces agreeing to participate were randomly allocated using a minimization process in a preprogrammed online algorithm [49] to either (1) the active intervention condition comprising the Helipad program or (2) an active control condition (an online time-matched standard psychoeducation program). The minimization approach to randomization [50] was used to balance across conditions on the following key workplace characteristics: (1) workplace type (office based vs non–office based), (2) organization size <200 vs ≥200), and location (the Australian Capital Territory, New South Wales, or Victoria vs other Australian state or territory). However, after the allocation of 20 organizations, we noticed that there was an issue with the minimization programming, which failed to account for stratification of factors, such that a significant imbalance in larger (≥200) organizations had been assigned to the control condition. To rectify this with a feasible solution, we asked a researcher independent of the trial to perform a manual minimization procedure based on workplace type and organization size, to improve the balance between conditions on these key strata. Each time a new workplace was recruited into the trial, the external researcher was notified via email by the trial manager of the workplace type and size, and if the relevant stratum had <50% intervention, the workplace would be allocated to the intervention, and if the relevant stratum had >50% intervention, then it would be allocated to the control. If the stratum had a 50% split at the time of minimization, then the external researcher would then use a random method of flipping a coin (ie, heads=intervention and tails=control) to determine the allocation.

### Employee Email Invitations

After allocation to a condition, we provided workplaces with an email with an embedded link to their assigned study recruitment website. We asked the key representative from each organization to arrange to send out invitations to all their employees to increase the ecological validity of the study given the expected future use of the program in workplaces.

Email text was customizable, with 13 organizations (17 workplaces) customizing the text to suit their workplace and 3 organizations (7 workplaces) choosing to send information to staff in a different form, such as an emailed staff newsletter. One organization (5 workplaces) elected to deliver the program via their usual education learning platform. They asked employees to register and then delivered the link to the program via their education learning platform to registered employees. Customized changes to text were typically made to provide further context and confirm the organization’s endorsement of the study. An example of a typical email invitation is displayed in Box 1.1 in Multimedia Appendix 1.

### Participant Recruitment and Informed Consent

Participants were recruited via direct invitation emails from their workplace. Employees who clicked on the link provided by their employers were taken directly to the information and consent online page, which provided written information about the study and a downloadable PDF of the same information.

### Participant Eligibility

Participants read an online information page and were required to confirm that they met the following criteria: (1) be an employee in a participating Australian workplace; (2) be aged ≥18 years or over, living in Australia, and fluent in reading and understanding English; and (3) have access to a device (desktop, laptop, tablet, and/or smartphone) and a reliable internet connection. If participants did not meet the eligibility criteria, they were directed to a page with a list of relevant help seeking resources.

### Patient and Public Involvement

As described earlier (Interventions section), the intervention was developed using a thorough co-design process facilitated by lived experience researchers (AG and CH) using iterative participatory methods [43]. A lived experience research officer was attached to the project to provide lived experience perspectives on the design and implementation of the trial, and the trial manager also identified as a lived experience researcher.

### Data Collection

Participants completed a brief pretest survey (10 min), followed immediately by access to their workplace-assigned program (approximately 20 min), after which they clicked immediately to the posttest survey (10 min). Participants in the intervention condition were then invited to participate in a follow-up interview to discuss their experiences. The results of these interviews (n=16) will be explored in detail in a planned paper on implementation. A final brief follow-up survey (10 min) was sent to participants via email at 6 months as outlined below.

### Participant Email Registration

Participants were required to enter an email in the pretest survey, which was encouraged in the information sheet to be a personal email, if possible, primarily to avoid participant loss if they changed workplaces during the 6-month follow-up period. We elected to collect emails only and avoided the need for participants to create an account to streamline access to the programs and maintain security and confidentiality while allowing participants to recommence their program if necessary.

### Email Reminders

The surveys and the program were expected to be completed in a single session; however, participants who discontinued any part of the program early were sent an email reminder to complete the program. This reminder email was sent at 48 hours if they discontinued any part of the pretest, program, or posttest survey and at 1 week for the 6-month follow-up survey. A unique link was provided in this email for the participant to click and continue at the same point in the survey or program that they left.

### Blinding

The trial was triple blinded. Participants were blinded as far as possible to whether they received the intervention or the active control condition—they were informed that their workplace was randomized to receive 1 of 2 programs without specifying which one they received (1) an enhanced interactive mental health education program or (2) a standard written psychoeducation program. We did not provide information on the hypothesis or the expected effectiveness of either program. Assessments were also blinded as far as possible as they were completed online by the participants themselves and were self-reported. Workplaces were also not informed of their assigned program to avoid this information inadvertently influencing outcomes. The researcher who performed the statistical analyses was not involved in the design or management of the study and was also blinded to condition, with the groups marked as “Condition X” and “Condition Z” in the data file. The statistician followed a prespecified statistical analysis plan, described in the protocol paper [39].

### Measures

#### Assessment Time Points

Demographic characteristics were collected at pretest; the primary outcome was collected at pretest, posttest, and 6-month follow-up; and the secondary outcomes were collected at pretest, posttest, and 6-month follow-up (help seeking attitudes, stigma, and mental health literacy) or pretest and follow-up only (help seeking behavior, mental health symptoms, quality of life, and functioning). We also collected data on acceptability, appropriateness, and feasibility at posttest, and adverse events at posttest and the 6-month follow-up.

#### Demographic Characteristics

The following demographic characteristics were collected at pretest: gender (male, female, nonbinary, different term—please specify), age (18‐25, 26‐35, 36‐45, 46‐55, 56‐65, and ≥66 y), language spoken at home (English only, English and another language, and another language only), ethnicity (free text: “How would you describe your ethnicity?”), level of education (primary school, some secondary school or year 10 equivalent, year 12, certificate level I-IV, diploma or associate degree, bachelor’s degree, graduate diploma or graduate certificate, master's degree, and doctoral degree), employment status (full-time, part-time or casual, unemployed, and not currently working due to studying or maternity leave), and work industry (full list of options is available in Multimedia Appendix 1). All demographic survey items included a “prefer not to answer” option. Ethnicity in Australia is complex [51]; thus, after discussion with the research team, we decided to deliberately leave the question on ethnicity as an open-ended question, to allow participants to self-identify. These were then coded as per the 9 classification ethnicity groups in Australia [52], then categorized into the 3 most prevalent categories: “Australian,” “North-West European/Caucasian,” “Asian,” with “Other.” Multimedia Appendix 1 presents a full description of the method for coding ethnicity.

#### Help Seeking Intentions (Primary Outcome)

Help seeking intentions were measured by the General Help-Seeking Questionnaire (GHSQ) intentions scale [53], which asks respondents’ intended likelihood to seek help from a list of help seeking sources *“*if you were having a personal or emotional” problem. We combined nonprofessional, informal sources into a single item (ie, “Partner, friend, parent, other relative/family member”) and added “Employee Assistance Programs*”* and “Internet-based treatment programs” to the potential sources of help. Responses were rated on a 7-point Likert-type scale, ranging from 1 (extremely unlikely) to 7 (extremely likely), with higher scores indicating greater intentions of seeking help from that source. A score for help seeking intentions from professional sources (mean of psychologist or counselor, psychiatrist, and family physician or GP) was created, with individual items also examined. At pretest, Cronbach α was 0.78.

#### Help Seeking Attitudes

Help seeking attitudes were measured using a brief updated 5-item version [54] of the Attitudes Towards Seeking Professional Psychological Help scale—short form [55]. Respondents were asked on a 4-point Likert-type scale, ranging from 0 (disagree) to 3 (agree), what extent they agree with statements about psychological treatment. We used a version [54] with updated wording, that is, “mental breakdown” was altered to “personal or emotional problems*.*” Items retained from the original measure were the positively worded items (ie, 1, 3, 5, 6, and 7) and based on factor loadings [56,57], for example, *“*If I was having personal or emotional problems, the first thing I would do is seek professional help*.*” Scores are summed (range 0‐15); higher scores indicate more positive attitudes toward seeking professional help. The brief 5-item (α=.72) [56] and 10-item scales display good psychometric properties [54,55,58]; pretest α=0.76.

#### Help Seeking Behavior

We used the Actual Help-Seeking Questionnaire [59] to assess help seeking behavior during the past 6 months. Respondents were asked, *“*have you sought advice or help in the past 6 months for a mental health problem?*”* Response options were “Yes”; “No, I did not seek advice or help”; or “No, I did not have any problems*.”* If “yes” was selected, respondents were presented with a list of potential sources of help. Potential sources of help or advice were matched to the GHSQ intentions scale, including professional and nonprofessional sources.

#### Mental Health Literacy

Items from the Depression Literacy [60] and Anxiety Literacy questionnaires [61] were used to develop a new measure of mental health literacy. The bespoke measure comprised 11 statements (5 Depression Literacy, 5 Anxiety Literacy, and 1 new item), and respondents indicated whether each statement is true, false, or don’t know. We added a new item on help seeking specifically for this study of “Avoiding professional help for my mental health is unlikely to have an effect on my health long-term” (false). Participants scored 1 point for each correct answer; higher scores indicated higher literacy (range 0‐11) [60,61]. Knowledge scales often do not report internal consistency, as it is typically important for the scale’s utility if respondents provide varied responses [62].

#### Mental Illness Stigma

We measured mental illness stigma by adapting the “stigma to others” scale from the Stigma and Self-Stigma Scales (SASS) for attitudes to mental health problems [63]. The adapted scale retained 4 of the 6 original SASS items that had acceptable factor loadings and did not duplicate other items. Minor changes to wording were also made, for example, we removed the word “suffering.” This left 4 items, including “Employees with a mental illness are less reliable than other employees” and “People with a mental illness are not really ill.” Items were rated on a 5-point scale, ranging from 0 (strongly disagree) to 4 (strongly agree). Higher mean scores indicate higher stigmatizing attitudes toward mental illness. The original SASS displays good validity and internal consistency (α=.71) [63], pretest α=0.81.

#### Depression and Anxiety Symptoms

Depression and anxiety symptoms were assessed using Patient-Reported Outcomes Measurement Information System (PROMIS)–short form scales (8 items each) [64] measuring symptom frequency in the past 7 days. Respondents rated the items on a 5-point scale, ranging from 1 (never) to 5 (always), which were summed for total scores (range 8‐40; higher scores=greater symptom severity). Example items include “I felt nervous” (anxiety) and “I felt unhappy” (depression). *T*-scores were also calculated using the available conversion tables [65]. Scales show strong validity; *r*=0.96 with full forms and *r*=0.80 to 81 with other established scales [64]; pretest anxiety (α=.94) and depression (α=.95).

#### General Psychological Distress

General psychological distress was assessed on the 5-item Distress Questionnaire (DQ5) [66]. Respondents rated the frequency they experienced distressing situations, thoughts, and feelings in the past 30 days on a 5-point scale, ranging from 1 (never) to 5 (always). Example items include “I felt hopeless” and “I found social situations upsetting.” Scores are summed for a total score (range 5‐25), with higher scores indicating greater levels of psychological distress. Previous research has demonstrated high internal consistency (α=.86) for the DQ5 [66,67]; pretest α=0.87.

#### Quality of Life

We measured quality of life on the Recovering Quality of Life-10 [68], a mental health recovery measure. It comprises 11 items, with 10 assessing the frequency in the past week of thoughts, feelings, or activities related to mental health, including “I felt lonely.” Items were rated from 0 (none of the time) to 4 (most or all of the time); 4 negatively worded items are reverse scored, and the scores are summed for a total possible of 0 to 40, with higher scores indicating perceived higher quality of life. The final (11th) item measured the severity of physical health problems experienced by the respondent, including “problems with pain, mobility, difficulties caring for yourself or feeling physically unwell” on a 5-point scale, ranging from 4 (no problems) to 0 (very severe problems). The scale has been validated (α=.87‐.92 [68,69]; pretest α=.87).

#### Work Productivity and Activity Impairment

We used the Work Productivity and Activity Impairment Questionnaire [70] to assess health-related impact on work and daily activities. We altered the scale to days during the past month (ie, 30 d), the visual analog scale to 1 to 11 (the survey platform was unable to capture the data if “0” was selected), and removed the first question on employment. The 5 questions asked participants to rate over the past 30 days: the number of days (1) missed due to health problems, (2) missed for other reasons, and (3) worked. They were also asked to rate the past 30 days on a 1‐ to 11-point visual analog scale: the (4) degree their health affected productivity while working and the (5) degree that their health problems affected their productivity in regular unpaid activities. We generated outcomes of *work productivity loss* (percentage overall work impairment) and *activity impairment* (percentage of regular daily [nonwork] activity impairment due to health) [71] for this study. Multimedia Appendix 1 presents full calculations to create these outcomes. Previous research has demonstrated validity for the original scale [72].

#### Adverse Events (Harms) and Usage Data

We collected broad data on participants’ experience of adverse events [73] by asking at posttest and follow-up, *“*did you have any unexpected or negative experiences that might be related to completing this program*?* (yes/no).” We also included an open-ended question for the participants to briefly describe what happened. Usage data such as time spent on each page were intended to be collected; however, we found after the trial that accurate data from Google Analytics could not be obtained by the web development team due to the complexity of the platform setup (ie, unique web links for each site).

### Quality Assurance and Monitoring

The clinical trial coordinator (AG) and the research officer (CH) were responsible for the day-to-day administration of the research, supervised by the primary investigator (PJB). This core team met regularly to discuss the research, weekly during the recruitment phase, and then fortnightly after that. A broader team meeting was also held monthly during recruitment to provide opportunities for input into the trial methods. Recruitment was monitored closely. Updates on recruitment sites were provided to the ANU HREC monthly to ensure they were kept informed of trial sites in case of issues or complaints.

### Protocol Deviations

In addition to the issue with minimization (outlined earlier), a deviation occurred regarding the delivery of the 6-month email that was sent to participants. First, a planned update to the online platform at the end of February 2025 disrupted the PostMark survey schedule. This meant that no 6-month reminders were sent for a period of 41 days. After this was detected, 35×6-month invitations were sent manually, and the postmark schedule was restored for the remaining surveys. A second issue identified at this time was that 6-month reminder emails had not been programmed for participants who had only partly completed the presurvey (ie, they had not finished the program and postsurvey). To rectify this, we examined the data that had been downloaded from the presurvey and identified all participants who had answered items on the last 2 measures (Work Productivity and Activity Impairment Questionnaire and Recovering Quality of Life) in the presurvey or started the program or the postsurvey but who had not completed the full program (ie, including the postsurvey). To preserve the intention to treat nature of the trial, these participants were sent manual invitation emails to complete the 6-month follow-up survey. Therefore, 45 invitations were sent out later than 6 months. A total of 146 reminders were sent manually on a weekly basis after this was discovered (March 2025). Almost all participants (control: 57/62, 91.9%; mean 6.38, SD 0.63 mo; and intervention: 56/59, 94.9%; mean 6.30, SD 0.47 mo) responded to the follow-up survey between 6 and 7 months. There was no significant difference between conditions on time in months between pre- and 6-month surveys (*t*_119_=0.79, *P*=.43).

Minor errors in sending invitation emails during the trial also occurred. These included when one organization that had 2 separate locations was split into 2 conditions and the administrator accidentally emailed one of the location’s center heads (who was to distribute it to the staff in that condition) the incorrect email link. As soon as the error was detected, the administrator sent the correct email and link through with instructions to ignore the previous email. Thus, it is unlikely the incorrect email reached the employees in this group and expect minimal impact from this error. Invitation emails with broken web links were sent to one of the organizations that had multiple sites; however, again we expect this had minimal impact as the emails were recalled very quickly (within 11 min) and were not distributed.

### Outcomes and Data Analysis

#### Hypotheses

To investigate the study aims, we hypothesized the following.

##### Primary Hypothesis (Aim 1)

The primary hypothesis was as follows:

- H1: There will be a greater increase in help seeking intentions (professional sources) in the Helipad program condition relative to the control condition (standard psychoeducation only) from pretest to posttest (primary end point) and from pretest to 6-month follow-up (secondary end point).

##### Secondary Hypotheses (Aim 2)

The secondary hypotheses were as follows:

- H2: There will be a greater decrease in mental illness stigma and a greater increase in mental health literacy and help seeking attitudes in the Helipad program condition relative to the control condition (standard psychoeducation only) from pretest to posttest, sustained at the 6-month follow-up.
- H3: There will be a greater increase in professional help seeking behavior in the Helipad program condition relative to the control condition (standard psychoeducation only) for people with elevated symptoms of depression or anxiety from pretest to 6-month follow-up.
- H4: There will be a greater decrease in symptoms of mental ill-health and a greater increase in quality of life and functioning in the Helipad program condition relative to the control condition (standard psychoeducation only) from pretest to the 6-month follow-up.

As planned in our protocol [39], exploratory hypotheses for implementation and cost-effectiveness outcomes will be explored in detail in separate publications.

### Power

Power and sample size estimations were calculated using the primary outcome measure: the GHSQ intentions to seek help from professional sources. On the basis of a similar workplace trial (MATES), which was trialed in construction workplaces [74], we used a conservative Intraclass Correlation Coefficient (ICC) of 0.02 to estimate the design effect. We estimated an expected between-groups effect size for GHSQ scores to be 0.2 based on a randomized clinical trial for an online program to increase help seeking intentions for social anxiety disorder [75]. We also assumed an average cluster size of between 25 and 35 and a conservative rate of 15% attrition in participant numbers from pre- to posttest based on previous workplace trials [76] and expectations that participants would complete the program and the pretest and posttest surveys in one session.

Our original power calculation, based on detecting an effect of *d* of 0.2 between intervention and comparison arms with 80% power using a type I error level of 0.05 (2-tailed), required recruiting between 30 and 36 workplaces (assuming 25 to 35 per workplace) and a minimum of 900 employees. After reaching our target number of workplaces, we found the number of participants per workplace was much lower than expected. Therefore, we revised our power calculation, aiming to recruit a greater number of workplaces to account for the lower number of participants in each workplace. Using the original parameters (ICC=0.02, *d*=0.2, 15% attrition, 80% power, type I error level=0.05 [2-tailed]), the new power calculation led to a revised required number of workplaces of 50, based on a cluster size of 17 (total N=850).

### Data Analysis

Outcomes were assessed at the individual level accounting for clustering by workplace. We used mixed-model repeated measures (MMRM) ANOVA as an intention-to-treat method, as this approach uses all available data. MMRM ANOVA accounts for within-participant variability and includes participants with missing data, under the missing-at-random assumption [77]. The within-groups factor was time point (pretest, posttest, and 6-mo follow-up), and the between-groups factor was trial condition (control and intervention). We used Cohen *d* to estimate between-group effect sizes based on observed means and SDs. To aid in interpretation, we reversed the formula for some outcomes so that a positive effect size indicated an advantage for the intervention condition. All analyses used SPSS version 29.0 for Windows (IBM Corporation).

### Primary Outcome (Aim 1)

Effectiveness of the program (H1) was assessed based on increased help seeking intentions from professional sources at posttest. Exploratory analyses also examined changes in help seeking intentions by condition from each help seeking source. We used MMRM ANOVA for continuous data accounting for clustering within each site, and all model assumptions were met.

### Secondary Outcomes (Aim 2)

Effectiveness for the secondary outcomes of increased mental health literacy and help seeking attitudes at posttest and mental illness stigma (H2) and mental ill-health, quality of life, and functioning at 6 months (H4) were also assessed using MMRM ANOVA adjusting for ICC. Effectiveness for the secondary outcomes (H3) of increased professional help seeking behavior at 6 months in those with elevated depression or anxiety (*T*-score ≥60 [65] at either pretest or 6-mo follow-up) was assessed using mixed-effects logistic regression.

Consistent with our protocol [39], we used linear mixed-effects regression models to investigate the effects of condition, time, and putative factors (age, gender, education, mental health literacy, stigma, depression, anxiety, and general psychological distress) that may moderate help seeking intentions from professionals. We included all 2- and 3-way interaction effects.

## Results

### Overview

**Figure 3.**
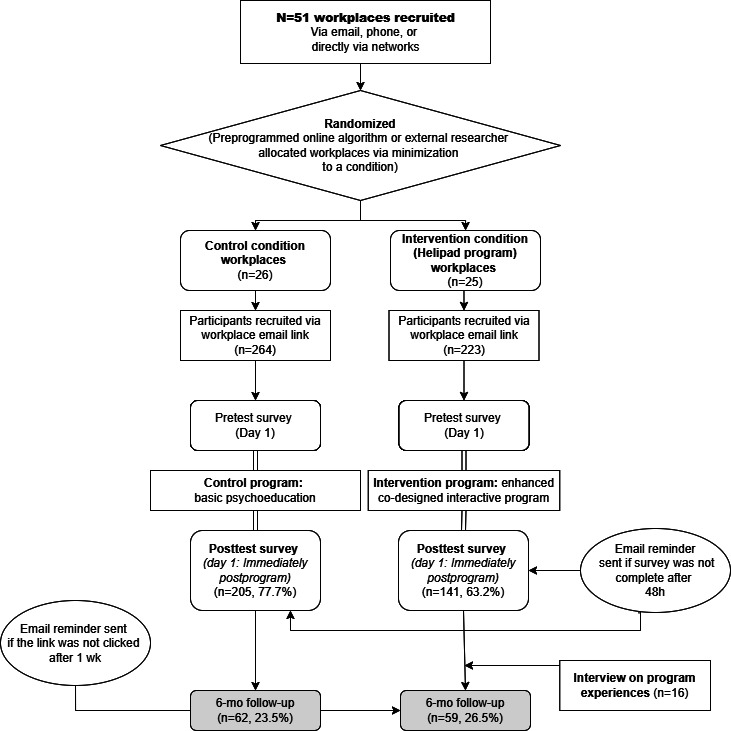
Cluster randomized controlled trial flowchart.

Figure 3 displays the CONSORT diagram of participant flow through the trial, and Table 1 presents the demographic information. From 614 employees (intervention: n=282 and control: n=332) who clicked the recruitment link and were screened on informed consent, 487 participated in the trial (intervention: n=223, 79.1%; and control: n=264, 79.5%). Overall retention rates for those who participated were 71.1% at posttest and 24.9% at follow-up.

**Table 1.** Demographic information for participants included in the study (N=487).

Characteristic	Control (n=264), n (%)	Helipad (n=223), n (%)	Total (N=487), n (%)
Age category (years)			
18‐25	25 (9.5)	24 (10.8)	49 (10.1)
26‐35	70 (26.5)	62 (27.8)	132 (27.1)
36‐45	75 (28.4)	58 (26.0)	133 (27.3)
46‐55	53 (20.1)	42 (18.8)	95 (19.5)
56‐65	34 (12.9)	33 (14.8)	67 (13.8)
66+	5 (1.9)	4 (1.8)	9 (1.8)
Prefer not to answer	2 (0.8)	0 (0.0)	2 (0.4)
Gender			
Male	58 (22.0)	55 (24.7)	113 (23.2)
Female	202 (76.5)	166 (74.4)	368 (75.6)
Nonbinary	2 (0.8)	1 (0.4)	3 (0.6)
Different term	1 (0.4)	1 (0.4)	2 (0.4)
Prefer not to answer	1 (0.4)	0 (0.0)	1 (0.2)
Language spoken at home			
English only	205 (77.7)	159 (71.3)	364 (74.7)
English and another language	57 (21.6)	57 (21.6)	114 (23.4)
Another language only	2 (0.8)	6 (2.7)	8 (1.6)
Prefer not to answer	0 (0.0)	1 (0.4)	1 (0.2)
Ethnicity			
Australian	97 (36.7)	58 (26.0)	155 (31.8)
North-West European/Caucasian	97 (36.7)	92 (41.3)	189 (38.8)
Asian	34 (12.9)	40 (17.9)	74 (15.2)
Other	36 (13.6)	33 (14.8)	69 (14.2)
Highest level of education			
High school or less	20 (7.6)	9 (4.0)	29 (6.0)
Certificate or diploma	81 (30.7)	47 (21.1)	128 (26.3)
Bachelor’s degree	75 (28.4)	81 (36.3)	156 (32.0)
Postgraduate degree/diploma	87 (33.0)	82 (36.8)	169 (34.7)
Prefer not to answer	1 (0.4)	4 (1.8)	5 (1.0)
Employment			
Full time	204 (77.3)	180 (80.7)	384 (78.9)
Part time or casual	58 (22.0)	42 (18.8)	100 (20.5)
Not working (eg, study or maternity leave)	2 (0.8)	0 (0.0)	2 (0.4)
Prefer not to answer	0 (0.0)	1 (0.0)	1 (0.2)
Industry			
Public services or administration	97 (36.7)	65 (29.1)	162 (33.3)
Health or social care	66 (25.0)	46 (20.6)	112 (23.0)
Teacher training or education	27 (10.2)	34 (15.2)	61 (12.5)
Science or pharmaceuticals	28 (10.6)	6 (2.7)	34 (7.0)
Recruitment or HR^a^	17 (6.4)	6 (2.7)	23 (4.7)
Hospitality or events	0 (0.0)	16 (7.2)	16 (3.3)
Engineering or manufacturing	3 (1.1)	12 (5.4)	15 (3.1)
Environment or agriculture	3 (1.1)	10 (4.5)	13 (2.7)
Business, consultancy, or management	6 (2.3)	5 (2.2)	11 (2.3)
Property or construction	0 (0.0)	7 (3.1)	7 (1.4)
Computing or IT	3 (1.1)	3 (1.3)	6 (1.2)
Law enforcement and security	1 (0.4)	3 (1.3)	4 (0.8)
Transport or logistics	3 (1.1)	1 (0.4)	4 (0.8)
Leisure, sport, or tourism	1 (0.4)	2 (0.9)	3 (0.6)
Marketing, advertising, or PR^b^	3 (1.1)	0 (0.0)	3 (0.6)
Accountancy, banking, or finance	1 (0.4)	1 (0.4)	2 (0.4)
Charity and voluntary work	1 (0.4)	1 (0.4)	2 (0.4)
Creative arts or design	1 (0.4)	1 (0.4)	2 (0.4)
Law	0 (0.0)	1 (0.4)	1 (0.2)
Media or digital	0 (0.0)	1 (0.4)	1 (0.2)
Retail or sales	1 (0.4)	0 (0.0)	1 (0.2)
Prefer not to answer	2 (0.8)	2 (0.9)	4 (0.8)

a HR: human resources.

b PR: public relations.

Table 2 presents the pre-test measures for participants included in the study. Full pretest measures for individual help seeking intentions (GHSQ) and behavior (Actual Help-Seeking Questionnaire) are presented in Table 1.1 in Multimedia Appendix 1.

**Table 2.** Pretest measures for participants included in the study (N=487).

Characteristic (potential range)	Total (N=487)	Helipad (n=223)	Control (n=264)
Help seeking intentions (GHSQ^a^, professionals—M score of psychologist or counselor, psychiatrist, GP^b^ (mean range 1‐7), mean (SD)	3.89 (1.53)	3.78 (1.42)	3.84 (1.48)
Help seeking attitudes (ATSPPH-SF^c^; total, range 0‐15), mean (SD)	9.63 (3.23)	9.78 (3.19)	9.70 (3.21)
Help seeking behavior (AHSQ^d^)—Any professional source ever previously sought (psychologist or counselor, psychiatrist, or GP), n (%)	83 (31.4)	64 (28.7)	147 (30.2)
Mental illness stigma (SASS^e^; mean, range 0‐4), n (%)	2.57 (2.51)	2.68 (2.73)	2.62 (2.61)
Mental health literacy (Combined D-lit^f^/A-lit^g^; total range 0‐11), n (%)	7.63 (2.06)	7.35 (2.21)	7.50 (2.13)
Depression (PROMIS^h^; total, range 8‐40), n (%)	16.43 (7.34)	16.80 (6.74)	16.60 (7.07)
Depression (PROMIS) *T*-score, n (%)	52.69 (9.40)	53.55 (8.29)	53.08 (8.91)
Anxiety (PROMIS; total, range 8‐40), n (%)	17.63 (7.05)	18.18 (6.93)	17.88 (6.99)
Anxiety (PROMIS) *T*-score, n (%)	54.57 (9.32)	55.46 (8.86)	54.98 (9.12)
General psychological distress (DQ5^i^; total, range 5‐25), n (%)	11.04 (4.54)	11.14 (4.05)	11.09 (4.32)
Work productivity loss (WPAI^j^; %; range 0‐100), n (%)	28.32 (26.30)	26.22 (22.61)	27.36 (24.67)
Activity impairment (WPAI; %; range 0‐100), n (%)	29.61 (27.25)	28.91 (25.71)	29.29 (26.53)
Quality of life (ReQoL^k^; total, range 0‐40), n (%)	27.86 (7.45)	27.73 (6.95)	27.80 (7.22)
Quality of life (ReQoL) physical health rating (range 0‐4)^l^, n (%)	3.11 (0.89)	3.12 (0.83)	3.12 (0.86)

a GSHQ: General Help-Seeking Questionnaire.

b GP: general practitioner.

c ATSPPH-SF: Attitudes Towards Seeking Professional Psychological Help scale—short form.

d AHSQ: Actual Help-Seeking Questionnaire.

e SASS: Stigma and Self-Stigma Scales.

f D-Lit: Depression Literacy Questionnaire.

g A-Lit: Anxiety Literacy Questionnaire.

h PROMIS: Patient-Reported Outcomes Measurement Information System.

i DQ5: Distress Questionnaire-5.

j WPAI: Work Productivity and Activity Impairment Questionnaire.

k ReQoL: Recovering Quality of Life-10.

l Lower scores indicate poorer physical health.

### Attrition

We assessed attrition based on the absence of completing the primary outcome from the survey at posttest and follow-up. A chi-square test (N=487; *χ*^2^_1_=10.4; *P*=.001) indicated fewer intervention condition participants (n=137, 61.4%) completed the primary outcome measure at the posttest survey than the control (n=198, 75%). However, no difference was found at the 6-month follow-up assessments (N=487*; χ*^2^_1_=0.03; *P=*.91). We conducted a multivariate logistic regression to examine whether any factors, including condition, demographic variables, and depression and anxiety symptoms at pretest, were associated with attrition on the primary outcome at the 6-month follow-up; however, there were no significant predictors (see Table 1.2 for full results in Multimedia Appendix 1).

### Primary Outcome

Table 3 displays the observed means and SDs for the primary and secondary outcomes. Means marked in bold indicate where there were significant between-group effects for primary and secondary outcomes at posttest or 6-month follow-up based on the MMRM ANOVA models showing significant time×condition interaction effects that reflect greater change from pretest in one condition compared to the other.

**Table 3.** Observed means and SDs for primary and secondary outcomes.

Condition	Time point					
	Pretest		Posttest		6-mo follow-up	
	Participants, n	Score, mean (SD)	Participants, n	Score, mean (SD)	Participants, n	Score, mean (SD)
Primary outcome						
Help seeking intentions (professional—mean score of psychologist or counselor, psychiatrist, GP^a^; potential range 1‐7)						
Control	260	3.89 (1.53)	198	4.12 (1.48)	54	4.09 (1.35)
Helipad	219	3.84 (1.48)	137	4.41 (1.31)^b^	47	4.38 (1.17)
Secondary outcomes						
Help seeking behavior (AHSQ^c^), n (%)—any professional source sought (psychologist or counselor, psychiatrist, or GP)						
Control	264	83 (31.4)	—^d^	—	62	16 (25.8)
Helipad	223	64 (28.7)	—	—	59	15 (25.4)
Help seeking behavior (AHSQ), n (%)—any professional source sought (psychologist or counselor, psychiatrist, or GP) for those with elevated PROMIS^e^ anxiety or depression symptoms (% meeting cutoff of *T*-score ≥60) at pretest or 6 mo						
Control	96	47 (49.0)	—	—	25	12 (48.0)
Helipad	83	37 (44.6)	—	—	27	10 (37.0)
Help seeking attitudes (ATSPPH-SF^f^; total, potential range 0‐15)						
Control	263	9.63 (3.23)	204	10.14 (3.03)	53	9.78 (2.72)
Helipad	223	9.78 (3.19)	141	10.81 (3.16)^g^	51	10.50 (2.64)
Mental illness stigma (SASS^h^; mean, potential range 0‐4)						
Control	264	2.57 (2.51)	204	2.13 (2.28)	54	1.74 (2.13)
Helipad	223	2.68 (2.73)	141	2.05 (2.51)	51	1.98 (1.86)
Mental health literacy (combined D-lit^i^/A-lit^j^; total, potential range 0‐11)						
Control	264	7.63 (2.06)	204	8.32 (1.90)	54	8.35 (1.73)
Helipad	223	7.35 (2.21)	140	9.52 (2.16)^k^	51	8.69 (1.70)
Depression (PROMIS; total, potential range 8‐40)						
Control	262	16.43 (7.34)	—	—	54	15.13 (6.04)
Helipad	223	16.80 (6.74)	—	—	51	15.13 (6.40)
Anxiety (PROMIS; total, potential range 8‐40)						
Control	262	17.63 (7.05)	—	—	54	17.06 (6.77)
Helipad	223	18.18 (6.93)	—	—	51	17.25 (6.69)
Depression (PROMIS)—elevated symptoms (% meeting cutoff of *T*-score ≥60)						
Control	262	62 (23.7)	—	—	54	7 (13.0)
Helipad	223	47 (21.1)	—	—	51	7 (13.7)
Anxiety (PROMIS)—elevated symptoms (% meeting cutoff of *T*-score ≥60)						
Control	262	78 (29.8)	—	—	54	13 (24.1)
Helipad	223	65 (29.1)	—	—	51	14 (27.5)
Either anxiety or depression (PROMIS)—elevated symptoms (% meeting cutoff of T-score≥60)						
Control	264	89 (33.7)	—	—	54	16 (29.6)
Helipad	223	76 (34.1)	—	—	51	14 (27.5)
General psychological distress (DQ5^l^; total, potential range 5‐25)						
Control	262	11.04 (4.54)	—	—	54	10.28 (4.44)
Helipad	223	11.14 (4.05)	—	—	51	10.36 (4.10)
Work productivity loss % (WPAI^m^)						
Control	255	28.32 (26.30)	—	—	56	23.75 (21.36)
Helipad	216	26.22 (22.61)	—	—	54	29.27 (25.71)
Activity impairment % (WPAI)						
Control	259	29.61 (27.25)	—	—	57	25.61 (26.32)
Helipad	220	28.91 (25.71)	—	—	54	27.59 (24.80)
Quality of life (ReQoL^n^; total, potential range 0‐100)						
Control	264	27.86 (7.45)	—	—	53	29.40 (6.43)
Helipad	223	27.73 (6.95)	—	—	50	29.52 (7.16)

a GP: general practitioner.

b Significant change from pretest to posttest versus control; *t*_359.35_=−3.72; *P*<.001.

c AHSQ: Actual Help-Seeking Questionnaire.

d Not applicable.

e PROMIS: Patient-Reported Outcomes Measurement Information System.

f ATSPPH-SF: Attitudes Towards Seeking Professional Psychological Help scale—short form.

g Pretest to posttest versus control;* t*=−2.02; *P*=.045.

h SASS: Stigma and Self-Stigma Scales.

i D-Lit: Depression Literacy Questionnaire.

j A-Lit: Anxiety Literacy Questionnaire.

k Significant change from pretest to posttest versus control; *t*_381.89_=−6.52;* P*<.001.

l DQ5: Distress Questionnaire-5.

m WPAI: Work Productivity and Activity Impairment Questionnaire.

n ReQoL: Recovering Quality of Life-10.

The primary hypothesis (H1) examined help seeking intentions from professionals (psychologist or counselor, psychiatrist, and GP). The main effect for time was significant overall for help seeking intentions *(F*_2,185.44_=29.84; *P*<.001), with significant improvements for both conditions pretest to posttest *(t*_362.36_=7.35; *P*<.001) and to follow-up (*t*_120.99_=3.35; *P*<.001).

A significant interaction was found between condition and time (pretest, posttest, and 6-mo follow-up; *F*_2,185.44_=6.89; *P*=.001). Planned contrasts showed there was a greater increase for the Helipad condition compared with the control condition from pretest to posttest for professional help seeking intentions (*t*_359.35_=−3.72; *P*<.001), but the difference between the 2 conditions was not statistically significant at the 6-month follow-up (*t*_119.76_=−1.05; *P*=.30). The between-group effect size at posttest for the intervention compared with control for help seeking intentions based on observed data was *d*=0.21 (95% CI –0.01 to 0.42), and at the 6-month follow-up, it was *d*=0.23 (95% CI –0.16 to 0.62).

**Figure 4.**
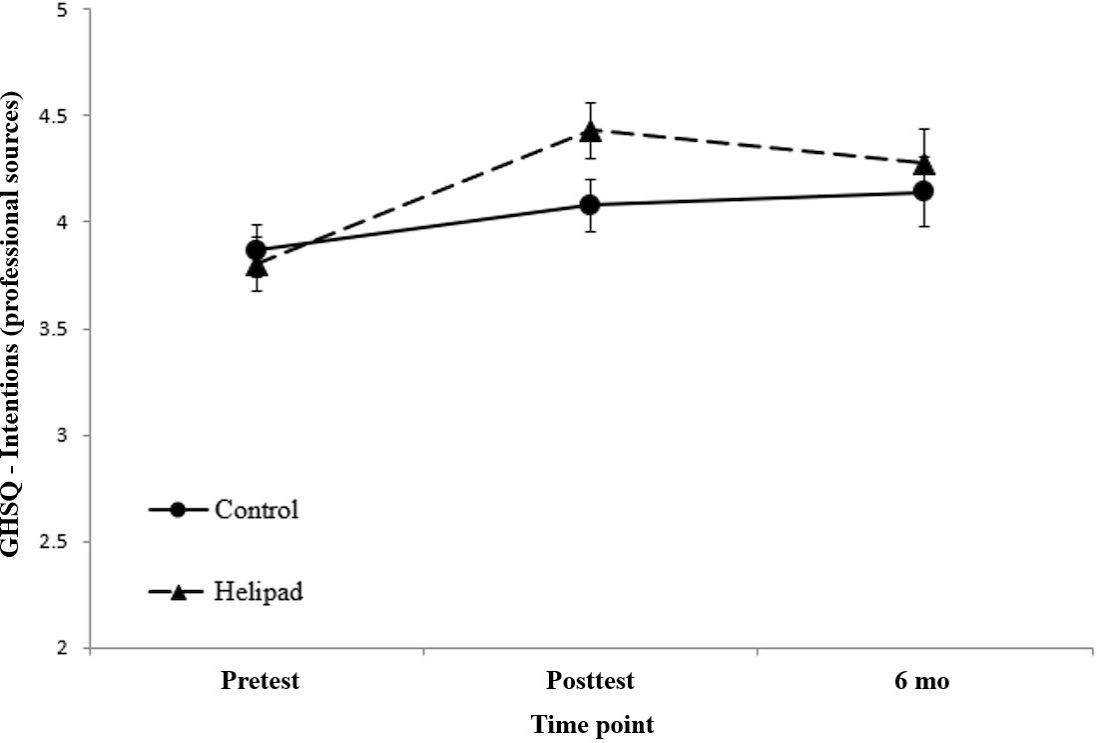
Estimated marginal means and SEs for help seeking intentions from professional sources (General Help-Seeking Questionnaire [GHSQ]) for each condition over time.

Figure 4 presents the estimated marginal means from the analysis of the primary outcome for each condition over time.

Exploratory analyses showed a significant interaction between conditions over the 3 time points (pretest, posttest, and 6-mo follow-up) for seeking help from a psychiatrist (*F*_1,186.10_=3.88; *P*=.02), GP (*F*_1,177.72_=13.00; *P*<.001), EAP (*F*_1,180.79_=4.20; *P*=.02), and phone helpline (*F*_1,173.64_=3.18; *P*=.04). Multimedia Appendix 1 presents the estimates (95% CI) of fixed effects from all MMRM models (Table 1.3 in Multimedia Appendix 1) and the full table of the observed means and SDs for the individual help seeking intentions (Table 1.4 in Multimedia Appendix 1).

### Secondary Outcomes

Table 3 displays the results for all secondary outcomes.

#### Help Seeking Attitudes

For H2, the main effect for time was significant for help seeking attitudes (*F*_2,192.65_=24.88; *P*<.001), with significant improvements for both conditions pretest to posttest (*t*_365.77_=5.93; *P*<.001) but not pretest to 6-month follow-up (*t*_121.14_=1.04; *P*=.30). There was no significant interaction over time between conditions for help seeking attitudes (*F*_1,192.65_=2.11; *P*=.12).

There was also a greater increase for the Helipad condition compared with the control condition from pretest to posttest for help seeking attitudes, and this was significant (*t*_363.99_=−2.02; *P*=.045), but again this difference between conditions was not statistically significant at the 6-month follow-up (*t*_119.27_=−0.26; *P*=.80). Effect sizes for help seeking attitudes were *d*=0.22 (95% CI 0.00-0.43) at posttest, and at the 6-month follow-up, it was *d*=0.27 (95% CI −0.12 to 0.66).

#### Mental Illness Stigma

Similarly, the main effect for time was significant for stigma *(F*_2,171.47_=7.00; *P*=.001), with significant improvements for both conditions from pretest to posttest (*t*_358.98_=−2.54; *P*=.01) but not pretest to follow up (*t*_112.34_=−0.84; *P*=.40). There was no significant interaction between conditions over time for stigma (*F*_1,171.47_=0.29; *P*=.97).

There was no significant interaction between conditions from pretest to posttest for stigma (*t*_352.16_=0.83; *P*=.93) or from pretest to 6-month follow-up (*t*_113.24_=−0.33; *P*=.74). The effect size for stigma was *d*=0.03 (95% CI −0.18 to 0.25), and at the 6-month follow-up, it was *d*=−0.12 (95% CI −0.50 to 0.26).

#### Mental Health Literacy

The main effect for time was also significant for mental health literacy (*F*_2,213.61_=86.00; *P*<.001), with significant improvements for both conditions pretest to posttest (*t*_394.80_=12.93; *P*<.001) and from pretest to 6-month follow-up (*t*_126.87_=4.30; *P*<.001). There was also a significant interaction between condition and time (pretest, posttest, and 6-mo follow-up) for mental health literacy (*F*_1, 213.61_=23.68; *P*<.001).

Planned contrasts showed that there was a greater increase in mental health literacy for the Helipad condition compared with the control condition from pretest to posttest (*t*_381.89_=−6.52; *P*<.001), but this difference was not statistically significant at the 6-month follow-up (*t*_125.42_=−0.68; *P*=.50). The between-group effect size at posttest for the intervention compared with control for mental health literacy was *d*=0.60 (95% CI 0.38-0.82), and at the 6-month follow-up, it was *d*=0.20 (95% CI −0.19 to 0.58).

#### Help Seeking Behavior

For H3, there was no significant interaction between condition and time (pretest to 6-mo follow-up) for those with elevated depression or anxiety symptoms (either at pretest or 6-mo follow-up) on help seeking behavior (any professional source sought, ie, psychologist or counselor, psychiatrist, and GP; odds ratio 0.76, 95% CI 0.21‐2.74; *P*=.67). This pattern was repeated when including all participants regardless of symptom levels (odds ratio 1.12, 95% CI 0.45‐2.78; *P*=.81). Tables 1.5 and 1.6 in Multimedia Appendix 1 display the number of participants reporting help seeking behavior from each individual source.

#### Mental Health Symptoms, Quality of Life, and Functioning

For H4, there was no significant interaction between condition and time (pretest to 6-mo follow-up) for depression symptoms (*F*_1,115.01_=1.31; *P*=.26), anxiety symptoms (*F*_1,110.96_=1.89; *P*=.17), general psychological distress (*F*_1,108.93_=0.85; *P*=.36), quality of life (*F*_1,107.81_=0.89; *P*=.35), and functioning (percentage of activity impairment; *F*_1,123.74_=0.71; *P*=.79), or functioning (percentage of work productivity loss*; F*_1,125.25_=1.51; *P*=.22).

#### Moderators of Intentions

The only significant moderator was general psychological distress measured using the DQ5 on help seeking intentions (*F*_2,178.34_=3.97; *P*=.02), which indicated that the effect of the intervention depended on participants’ distress levels at pretest. Those in the Helipad condition had improved help seeking intentions from professionals over time regardless of their pretest psychological distress levels, whereas in the control group, only those with high distress at pretest had improved intentions over time. This effect is illustrated in Figure 1.1 in Multimedia Appendix 1 using a binary indicator of low vs high distress on the DQ5 (0‐10=low distress; 11‐25=high distress).

### Adverse Events

A total of 15 people (control: n=9, 4.5%; and intervention: n=6, 4.3%) reported adverse events at posttest, and 3 people (control: n=2, 3.2%; and intervention: n=1, 1.7%) at follow-up, with no significant difference between conditions. At posttest for the control condition, 5 noted that the program brought up current distress or distressing events, and 4 reported that the program was text-heavy and not of benefit, or frustrating: “I found this program extremely frustrating. Huge paragraphs of text without any images or exercises to break it up*”* (control participant, posttest). For the Helipad condition, one mentioned they felt they were taking too long, and this made them feel anxious, while the other 5 focused on how the program made them reflect on their current distress:

*It made me realise that while I understood my anxiety [which is controlled through low dose medication, yoga and where needed tele counselling] exists, that it is at a much higher rate than I was appreciating...While not pleasant or expected it was a good reminder that I’m not alone and that I do have other options than keeping on keeping on.*

[Helipad participant, posttest]

At the 6-month follow-up, the 2 participants in the control group mentioned events that had happened to them at work during this time without specifically noting the program as a causative factor, whereas the Helipad participant mentioned that because of the program, they went to their GP but that it was not a helpful experience:

*I went to my GP about mental health because it was part of the program. Noting that it wasn’t a long appointment [i.e. about a mental health plan], I felt much worse afterwards. I probably needed to hear [some of] what she said, but I am not sure I’d ever speak to her about mental health again.*

[Helipad participant, 6-mo follow-up]

## Discussion

### Principal Findings

This study reports the primary and secondary outcomes of a randomized controlled trial to evaluate the impact of “Helipad,” a co-designed interactive program for increasing professional help seeking intentions in workplace employees. The program delivered psychoeducation tailored to Australian workers based on a co-design process that emphasized the need for better understanding of symptoms and treatments, interactive step-by-step directions for specific treatment pathways in Australia, guidance for promoting supportive nonstigmatizing workplaces, and equipping staff to respond appropriately to others who may be experiencing mental health symptoms. As presented in Table 3 (MMRM analyses) and Figure 4, the Helipad program was found to be effective for employees in workplace settings on the primary outcome of professional help seeking intentions compared with the control at the primary end point (immediate posttest), albeit with a small effect size. Although the effect size remained consistent at the 6-month follow-up, there was substantial attrition such that comparisons were underpowered, and differences between conditions were no longer significant. Compared with the control arm, which comprised written psychoeducation, the Helipad program also significantly improved mental health literacy and help seeking attitudes at posttest, but not mental illness stigma. The study was not powered to detect changes in help seeking behaviors, as only a proportion of participants would be expected to engage with services over the 6-month follow-up. Subsequently, no evidence was found that the Helipad program improved professional help seeking behavior; work and activity functioning; quality of life; or depression, anxiety, and general psychological distress symptoms (secondary outcomes) compared with the control at the 6-month follow-up. However, there were main effects of time for professional help seeking intentions, help seeking attitudes, mental illness stigma, and mental health literacy, indicating that both groups had significant improvements at posttest, with intentions and mental health literacy also significantly improved at the 6-month follow-up.

As hypothesized, the co-designed Helipad program was significantly more effective than basic psychoeducation at improving professional help seeking intentions from pre- to posttest in employees in a workplace setting. In contrast, previous online workplace programs have not found improvements in professional help seeking intentions albeit at longer follow-up time points, with one finding improvement in intentions to seek help from the internet at the 6-month follow-up [78], and the other finding an increase only in help seeking attitudes at posttest (3 mo) [79]. Although we did not find increases in professional help seeking behavior at 6 months, programs such as Helipad have the potential to equip workers with the knowledge they require to seek appropriate help when it is needed. Given that the effects on help seeking intentions were equivalent to the basic psychoeducation at 6 months, booster sessions [80], such as reminder emails, posters, or postcards at critical time points throughout the work year, may be useful. Furthermore, pairing Helipad with organizational programs to improve workplace culture and psychological safety may lead to more sustained changes.

Despite both Helipad and the active control program showing significantly improved help seeking intentions, attitudes, and mental health literacy at both the posttest and at 6 months, the Helipad program was superior to the control at posttest. Basic psychoeducation similar to that used in our control program has been found to be beneficial for improving help seeking intentions and attitudes [36] and mental health literacy in adults. However, in this trial, the Helipad program showed greater benefits. There are a range of potential reasons why the Helipad program performed better than the control. Helipad was co-designed to include information valued by people with lived experience of mental ill-health and other workplace stakeholders [43]. Helipad included specific content addressing care pathways and content that considered the role of the workplace in supporting mental health, so it may have been more effective for promoting help seeking and better targeted to both the local Australian context and the workplace setting. In addition, it specifically included a variety of content that was viewed as visually appealing and engaging by people with lived experience [81], such as videos, clickable diagrams, and interactive quizzes. Participants reporting adverse events noted that the control program was challenging to engage with and complete because of the lack of interactivity and images and the volume of written text. This finding suggests that engagement with Helipad may have influenced its effectiveness, although it should also be noted that survey attrition was higher in the Helipad condition.

Helipad was not better than standard psychoeducation at improving stigma, with significant improvements in both groups at posttest but neither at the 6-month follow-up. We expected the basic psychoeducation program to have a modest impact on stigma, but it was contrary to expectations that the Helipad condition did not perform better given the inclusion of videos of people with lived experience and the specific inclusion of myth-busting information around common misconceptions people may have around the experience of depression or anxiety [47]. Ramírez-Vielma et al [82] in their review of workplace stigma reduction programs, specifically noted that strategies involving lived experience “contact” has not been a commonly used strategy in workplace interventions and may not be more important than skills development in this context, particularly for recognizing and supporting co-workers who may be experiencing mental ill-health. Nevertheless, the finding contrasts with other evidence that contact interventions are more effective than psychoeducation [48], which may suggest that there is variability in the forms of contact and types of lived experience stories that might be optimal for addressing stigma.

Our analysis indicated that participant distress moderated program effectiveness. Those with high distress at pretest had increased help seeking intentions from professionals at posttest regardless of condition, whereas for those with low distress, only those who were in the Helipad condition had improved help seeking intentions at posttest. This suggests that those with low distress may have struggled to engage with the control program, whereas those with higher distress may have had greater motivation to engage with either program. This finding implies that the Helipad program may be effective in the prevention space to increase knowledge and help seeking intentions and attitudes for delivery to universal to indicated populations [27]. In contrast, basic written psychoeducation, while somewhat effective, may only engage those with a perceived need for support due to higher psychological distress. It is thought that participants with greater symptoms may experience greater motivation to engage in a program [45].

The Helipad program was also beneficial for increasing intentions to seek help from GPs, EAP, psychiatrists, and phone helplines, whereas no significant effects were found for these help sources in the control condition. The Helipad program included videos of a GP, EAP, and psychologist and included targeted information specifically about different types of health professionals, such as accessing GPs and EAPs, whereas the control provided no information on types of health professionals. Given Helipad was able to improve intentions to seek help from EAPs, these findings are particularly important in a workplace setting, where the use of EAP is low (estimated as accessed by only 6 % of employees) and thought to be driven by a lack of knowledge about EAPs and what they do [83].

### Limitations and Strengths

The strengths of this study include the rigorous trial design that accounted for clustering, use of validated outcome measures, the large sample size, an active control group and triple blinding, and the wide range of workplace settings that participated. Previous trials have developed effective programs for specific settings such as health care [32] and construction [33]. While small numbers in each workplace precluded analysis by workplace type, it is encouraging that the Helipad program was effective in this study including a range of settings and, given the findings, could be delivered in multiple workplaces, including those identified as being suited for programs on mental health promotion [84].

The primary limitation of the trial is that recruitment at each site fell short of targets, but this was compensated for by increased sites. Different methods used across organizations to recruit staff impacted engagement at each organization—for example, inclusion of the trial invitation in newsletters had poor uptake by staff, while direct emails and support from management teams led to higher uptake. Individuals and workplaces who engaged with the trial may not have been representative of the population of Australian workers, with nonresponders potentially having less personal interest in mental health. Although we exceeded our initial target for recruiting workplaces, it was challenging to recruit organizations for the purposes of the research trial, as organizational participation required a high degree of engagement from HRs or management teams. We note that during the trial, organizations that had leaders who understood the importance of mental health to their workplace were more likely to engage. These challenges may be less evident if the program was delivered without the research component. A further limitation was that the Google Analytics data on usage, such as time spent on each page, were unable to be obtained; however, given it was a single-session program, these data are unlikely to be as meaningful for determining engagement as programs delivered over longer periods.

Attrition levels were somewhat higher than expected, particularly at follow-up, where only one-quarter of participants completed the survey. Attrition may have been related to low perceived need among well individuals or due to participants leaving their organizations. Other potential reasons could include the late batch of 6-month reminders, the low intensity of the intervention, that is, participants may have forgotten the intervention by 6 months. In addition, the reminder email was minimal in design (see Box 1.2 in Multimedia Appendix 1) and may not have been sufficient to remind participants of the program they completed. Although our MMRM models accounted robustly for missing data [77], attrition reduced statistical power, and findings may have been influenced by differential attrition within certain groups.

The error in minimization presents a minor threat to internal validity due to the deviation in the protocol from a computerized system to a process with manual oversight. We sought to mitigate some of the potential impacts of this deviation by ensuring that the revised minimization process maximized balance by correcting the inaccuracies of the computerized system and by having a researcher external to the project team oversee the allocation process. There were a range of potential secondary outcomes that were not measured, particularly changes in comfort or confidence about providing help to others, which may be important to assess in future trials. The follow-up period and relatively low prevalence of mental health problems in the sample may have limited our ability to detect changes in service use behaviors and in mental health outcomes. Although typical for internet-based programs [85], the study sample being predominantly female and highly educated may limit the representativeness of the findings. Finally, adaptation of the intervention for international audiences may require some modification and further evaluation, ensuring that the content accounts for potential differences in healthcare systems, workplace cultures, stigma, and access to mental health services.

### Conclusions

This randomized controlled trial tested the effectiveness of a co-designed online help seeking program on increasing professional help seeking intentions in employees using naturalistic delivery across diverse workplace settings. The co-designed Helipad program was effective, significantly improving professional help seeking intentions compared with the active control program at the primary end point (immediate posttest), albeit with a small effect size. Participants using the Helipad program also had significantly improved mental health literacy and help seeking attitudes at posttest. However, our test of long-term outcomes can only be considered preliminary due to high attrition rates (~75%) at 6 months, with between-group differences no longer being significant and with no demonstration of behavior change. Longer follow-up time frames with a larger or more at-risk cohort would be needed to demonstrate impacts on help seeking behaviors, as a minority of participants were experiencing clinically significant symptoms that would warrant mental health service use. Consequently, the outcomes suggest that while implementation of the program may enhance knowledge and intentions to seek help, extended engagement through booster sessions or adjunctive organization-level components may be needed to support behavioral change. Further investigation of the cost-effectiveness of the program and considerations for implementation will be described in subsequent publications. This was the first trial of a co-designed intervention to improve mental health help seeking in general workplaces, with previous trials focusing primarily on specific high-risk work environments. Findings suggest that workplace programs that can be delivered at scale have strong potential to provide broad education for employees on mental health and how to seek help. These effects may lead to more appropriate engagement with professional mental health care, which may prevent poor mental health outcomes, increase productivity, and promote employee well-being.

## Supplementary material

10.2196/89215 Multimedia Appendix 1 Supplementary material.

10.2196/89215 Checklist 1 CONSORT-eHEALTH checklist (V 1.6.1).

10.2196/89215 Checklist 2 CONSORT 2025 checklist.

10.2196/89215 Checklist 3 TIDieR checklist.

## Abbreviations

ACT: Australian Capital Territory
CBT: cognitive behavioral therapy
CONSORT: Consolidated Standards of Reporting Trials
DQ5: Distress Questionnaire-5
EAP: employee assistance program
GHSQ: General Help-Seeking Questionnaire
GP: general practitioner
HR: human resources
MAGNET: Mental Health Australia General Clinical Trials
MMRM: mixed-model repeated measures
NHMRC: National Health and Medical Research Council
NIH: National Institutes of Health
PROMIS: Patient-Reported Outcomes Measurement Information System
RCT: randomized controlled trial
SASS: Stigma and Self-Stigma Scales
TIDieR: Template for Intervention Description and Replication
