# Supplementary material for: Effectiveness of a Brief Internet-Based Workplace Training Program to Support Help Seeking for Mental Ill-Health: Cluster Randomized Controlled Trial
Source: J Med Internet Res. 2026 Aug 7;28:e89215. doi: 10.2196/89215 (PMC13450809; doi:10.2196/89215)
Supplement: Supplementary file 3 [file jmir-v28-e89215-s001.docx]

# Multimedia Appendix 1: Supplementary file

**Box 1.1.** Example recruitment email invitation to employees sent from workplaces

*Dear <employees>,*

*We would like to invite you to participate in a voluntary research study evaluating an online program designed to change knowledge and beliefs around mental health in workplaces.*

*The study will take approximately 40 minutes to complete. This includes a short survey (10 mins), followed by a brief program (20 mins), and another survey (10 mins) to check up on how you’re doing after completing the program.*

*Please click the <link> or access the QR code below to participate.*

*<QR code>*

*Best wishes,*

*<workplace name>*

*On behalf of*

*The HeLiPaD project team*

*E:* [*helipad@anu.edu.au*](mailto:helipad@anu.edu.au)

*This study is conducted by researchers from The Australian National University (ANU), Black Dog Institute, and Deakin University and is supported by funding from the Mental Health Australia General Clinical Trials Network (#MRF2006296). The ethical aspects of this research have been approved by the ANU Human Research Ethics Committee (2023/053).*

## Work industry

Participant options for the demographic characteristic of work industry were as follows: business, consultancy or management; accountancy, banking or finance; charity and voluntary work; creative arts or design; energy and utilities; engineering or manufacturing; environment or agriculture; health or social care; hospitality or events; computing or IT; law; law enforcement and security; leisure, sport or tourism; marketing, advertising or PR; media or digital; property or construction; public services or administration; recruitment or HR; retail or sales; science or pharmaceuticals; teacher training or education; transport or logistics, or other (free text).

## Ethnicity coding

To capture maximal variation in ethnicity, if participants described themselves as ‘Australian’ or ‘English’ or ‘Anglo’ and any other ethnicity, this was coded as the other i.e., Australian and Chinese, was coded as ‘North-East Asian;’ Anglo/Australian=North-West European. If noted Australian only, it was coded as 'Australian.' The majority of participants described their ethnicity as Australian, with *n*=15 of these identifying as Aboriginal and Torres Strait Islander or First Nations peoples, or from a ‘Caucasian’ ‘Anglo’ or North-Western European background (e.g., England), with lesser numbers from Asian countries (‘South-East’, ‘North-East’, and ‘Southern and Central Asian’), and other ethnicities, including Southern and Eastern Europe (*n*=19), and People of the Americas (*n*=11).

Table 1.1 presents the pre-test measures for the individual help seeking intentions and help seeking behavior items for participants included in the study.

**Table 1.1.** Pre-test measures for participants included in the study (*N*=487)

|  | **Control**  (*n*=264) | | **Helipad**  (*n*=223) | | **Total**  (*N*=487) | |
| --- | --- | --- | --- | --- | --- | --- |
| **Characteristic** |  |  |  |  |  |  |
|  | ***12*** | ***SD*** | ***M*** | ***SD*** | ***M*** | ***SD*** |
| **GHSQ intentions** (primary outcome) |  |  |  |  |  |  |
| Help seeking intentions (professional sources: psychologist, psychiatrist, GP) | 3.89 | (1.53) | 3.78 | (1.42) | 3.84 | (1.48) |
| **GHSQ intentions - scale items** |  |  |  |  |  |  |
| Partner, friend, parent, other relative/ family member | 6.09 | (1.18) | 6.17 | (1.22) | 6.13 | (1.20) |
| Psychologist or counsellor | 4.36 | (1.83) | 4.43 | (1.80) | 4.39 | (1.82) |
| Psychiatrist | 3.01 | (1.71) | 2.79 | (1.62) | 2.91 | (1.67) |
| Family doctor/GP | 4.31 | (1.84) | 4.12 | (1.80) | 4.22 | (1.82) |
| Employee assistance program (EAP) | 3.80 | (1.73) | 3.75 | (1.86) | 3.78 | (1.79) |
| Phone help line (e.g. Lifeline) | 2.88 | (1.62) | 2.70 | (1.53) | 2.80 | (1.58) |
| Internet-based treatment program | 3.14 | (1.63) | 2.96 | (1.67) | 3.06 | (1.65) |
| I would not seek help from anyone | 2.56 | (1.72) | 2.69 | (1.87) | 2.62 | (1.79) |
| **AHSQ: Help seeking behavior** | ***n*** | **(%)** | ***n*** | **(%)** | ***n*** | **(%)** |
| *No, I did not have any problems* | 86 | 32.6 | 70 | 31.4 | 156 | 32.0 |
| *No, I did not seek advice or help* | 79 | 29.9 | 71 | 31.8 | 150 | 30.8 |
| *Yes* | 98 | 37.1 | 80 | 35.9 | 178 | 36.6 |
| *No answer* **^a^** | 1 | 0.4 | 2 | 0.9 | 3 | 0.6 |
| Help seeking behavior (AHSQ) - *Any professional source ever previously sought (psychologist, psychiatrist, GP)* | 83 | 31.4 | 64 | 28.7 | 147 | 30.2 |
| **AHSQ behavior individual items** |  |  |  |  |  |  |
| Partner, friend, parent, other relative/ family member | 77 | 29.2 | 69 | 30.9 | 146 | 30.0 |
| Psychologist or counsellor | 59 | 22.3 | 48 | 21.5 | 107 | 22.0 |
| Psychiatrist | 11 | 4.2 | 8 | 3.6 | 19 | 3.9 |
| Family doctor/GP | 53 | 20.1 | 41 | 18.4 | 94 | 19.3 |
| Employee assistance program (EAP) | 22 | 8.3 | 19 | 8.5 | 41 | 8.4 |
| Phone help line (e.g. Lifeline) | 8 | 3.0 | 3 | 1.3 | 11 | 2.3 |
| Internet-based treatment program | 3 | 1.1 | 6 | 2.7 | 9 | 1.8 |
| Other | 6 | 2.3 | 3 | 1.3 | 9 | 1.8 |

Note: **^a^** These missing data apply to the below individual items, i.e., control=1 (0.4%), Helipad=2 (0.9%), total

***Work Productivity and Activity Impairment (calculations)***

To create the scales used in the present study, four main outcomes were generated *absenteeism* (percentage of work time missed due to health) Q1/(Q1+Q3), *presenteeism* (percentage of impairment experienced while at work due to health) Q4/10, *work productivity loss* (percentage overall work impairment i.e., combined absenteeism and presenteeism due to health) Q1/(Q1+Q3) + [(1-(Q1/(Q1+Q3)))*(Q4/10)], *activity impairment* (percentage of regular daily (non-work) activity impairment due to health) Q5/10, with all scores multiplied by 100 for final percentages (range=0-100). The VAS scale was recoded to 0-10 for calculations.

### Attrition

Table 1.2 presents the logistic regression analysis for attrition, which indicated that there were no factors associated with attrition at 6-month follow-up.

**Table 1.2.** Logistic regression model for attrition (did not complete primary outcome at 6-month follow-up (*N*=487)

|  | **S.E.** | **Wald χ^2^** | **OR** | ***CI*** | ***P*** |
| --- | --- | --- | --- | --- | --- |
| *Intercept* | 0.65 | 5.5 4.628 | 4.63 |  |  |
| *Gender (Male)* | 0.29 | 0.5 | 0.82 | (0.47-1.43) | .48 |
| **Age** |  |  |  |  |  |
| 18-35 years | 0.39 | 2.20 | 0.56 | (0.26-1.20) | .14 |
| 36-55 years | 0.37 | 2.48 | 0.56 | (0.27-1.15) | .12 |
| *(56+ years)* |  |  |  |  |  |
| **Education** |  |  |  |  |  |
| Certificate/diploma/Bachelor’s | 0.49 | 0.01 | 1.06 | (0.41-2.75) | .91 |
| Higher degree | 0.50 | 0.36 | 0.74 | (0.28-1.98) | .55 |
| *(High school or less)* |  |  |  |  |  |
| Employment *(Full-time)* | 0.33 | 3.40 | 1.84 | (0.96-3.50) | .07 |
| Language *(English only)* | 0.29 | 2.11 | 1.52 | (0.86-2.67) | .15 |
| Depression symptoms | 0.03 | 0.90 | 1.02 | (0.98-1.08) | .34 |
| Anxiety symptoms | 0.03 | 0.01 | 1.00 | (0.95-1.05) | .94 |
| Condition (*Control*) | 0.23 | 0.01 | 0.98 | (0.62-1.53) | .92 |

Note: *(Reference category).*

# Mixed models repeated measures analyses

Table 1.3 presents the results of the MMRM ANOVAs for the primary outcome (H1) of help seeking intentions from professionals (psychologist, psychiatrist, GP), and the secondary outcomes of help seeking attitudes, mental illness stigma, and mental health literacy.

**Table 1.3.** Estimates (95% CI) of fixed effects from MMRM models

|  | **Effect (95% CI)** | | | | |
| --- | --- | --- | --- | --- | --- |
| **Outcome** | Condition  (Helipad vs Control) | Timepoint  (post-test vs pre-test) | Timepoint  (6-months vs pre-test) | Interaction  (Helipad vs Control at post-test) | Interaction  (Helipad vs Control at 6-months) |
| Help seeking intentions (professional – mean) | 0.06 (-0.29, 0.42) | **0.63^a^** (0.46, 0.80) | **0.47^a^** (0.19, 0.75) | **-0.42^a^** (-0.64, 0.20) | -0.20 (-0.58, 0.18) |
| *Partner, friend, parent, other relative/ family member* | -0.10 (-0.37, 0.17) | -0.02 (-0.14, 0.11) | 0.01 (-0.23, 0.24) | -0.04 (-0.20, 0.13) | 0.07 (-0.25, 0.39) |
| *Psychologist or counsellor* | -0.11 (-0.59, 0.37) | **0.51^a^** (0.31, 0.72) | **0.59^a^** (0.26, 0.92) | -0.25 (-0.52, 0.02) | -0.40 (-0.85, 0.05) |
| *Psychiatrist* | 0.21 (-0.12, 0.54) | **0.54^a^** (0.35, 0.73) | **0.38^b^** (0.05, 0.72) | **-0.36^b^** (-0.61, -0.10) | -0.19 (-0.65, 0.28) |
| *Family doctor/GP* | 0.16 (-0.27, 0.58) | **0.81^a^** (0.61, 1.01) | 0.33 (-0.03, 0.70) | **-0.67^a^** (-0.94, -0.41) | 0.05 (-0.45, 0.55) |
| *Employee assistance program (EAP)* | 0.03 (-0.38, 0.44) | **0.46^a^** (0.27, 0.66) | **0.39^b^** (0.04, 0.74) | **-0.35^b^** (-0.61, -0.09) | 0.10 (-0.38, 0.59) |
| *Phone help line (e.g. Lifeline)* | 0.18 (-0.13, 0.49) | 0.42 (0.23, 0.61) | **0.33^b^** (0.05, 0.60) | **-0.31^b^** (-0.56, -0.06) | -0.16 (-0.54, 0.22) |
| *Internet-based treatment program* | 0.11 (-0.25, 0.47) | **0.43^a^** (0.21, 0.66) | **0.56^a^** (0.19, 0.93) | **-0.30^b^** (-0.59, -0.01) | -0.16 (-0.67, 0.34) |
| *I would not seek help from anyone* | -0.12 (-0.53, 0.29) | **-0.53^a^** (-0.74, -0.32) | **-0.46^b^** (-0.85, -0.06) | 0.13 (-0.14, 0.41) | 0.55 (0.01, 1.08) |
| Help seeking attitudes (ATSPPH-SF) | -0.40 (-1.19, 0.39) | **1.06^a^** (0.71, 1.41) | 0.27 (-0.25, 0.79) | **-0.47^b^** (-0.93, -0.01) | -0.09 (-0.81, 0.63) |
| Mental illness stigma (SASS) | -0.17 (-1.22, 0.88) | **-0.30^b^** (-0.54, 0.07) | -0.18 (-0.61, 0.25) | 0.01 (-0.30, 0.32) | -0.10 (-0.69, 0.49) |
| Mental health literacy (Combined D-lit/A-lit) | 0.26 (-0.37, 0.89) | **2.10^a^** (1.78, 2.41) | **0.89^a^** (0.48, 1.30) | **-1.40^a^** (-1.81, -0.97) | -0.19 (-0.76, 0.37) |

Note: **^a^** *P*<.001; **^b^** *P*=.05

# Planned contrasts

The results indicated that the control and the Helipad condition had differing independent impacts on help seeking intentions, mental health literacy, help seeking attitudes, and stigma. These contrasts are outlined below.

## Primary outcome

### Help seeking intentions

Planned contrasts showed that each condition showed a significant increase in professional help seeking intentions from pre-test to post [control *t*(352.81)=2.91, *P*=.004); Helipad [*t*(363.09)=7.33, *P*<.001)], and from pre-test to 6-month follow-up [control *t*(118.75)=2.07, *P*=.04); Helipad [*t*(120.29)=3.34, *P*=.001)].

## Secondary outcomes

### Mental health literacy

Each condition demonstrated through the planned contrasts a significant increase in mental health literacy from pre-test to post-test [control *t*(371.44)=4.98, *P*<.001); Helipad [*t*(388.08)=12.77, *P*<.001)], and this was maintained from pre-test to 6-month follow-up [control *t*(123.94)=3.96, *P*<.001); Helipad [*t*(126.60)=4.75, *P*<.001)].

### Help seeking attitudes

Planned contrasts showed that each conditions showed a significant increase in help seeking attitudes from pre-test to post-test [control *t*(358.27)=3.90, *P*<.001); Helipad [*t*(367.21)=5.93, *P*<.001)], but not from pre-test to 6-month follow-up [control *t*(117.88)=0.71, *P*=.48); Helipad [*t*(120.24)=1.05, *P*=.30)].

### Stigma

Each condition showed a significant decrease through the planned contrasts in stigma from pre-test to post-test [control *t*(347.42)=-2.87, *P*=.004); Helipad [*t*(354.86)=-2.51, *P*=.01)], but not from pre-test to 6-month follow-up [control *t*(112.89)=-1.66, *P*=.10); Helipad [*t*(113.52)=-1.14, *P*=.26)].

### Intentions – individual scale items

We also investigated the individual items on the GHSQ scale. Table 4.4 presents the means and standard deviations at each time point with significant changes highlighted.

There was no significant interaction between condition and time (pre, post, 6-month follow-up) for help seeking intentions from a partner, friend, parent, other relative/family member [*F*(1, 177.08)=0.25, *P*=.78], psychologist [*F*(1, 197.04)=2.40, *P*=.09], internet treatment program [*F*(1, 175.67)=2.10, *P*=.13], or not seeking help from anyone [*F*(1, 186.91)=2.42, *P*=.09]. However, a significant interaction between conditions over time (pre, post, 6-month follow-up) was found for seeking help from a psychiatrist [*F*(1, 186.10)=3.88, *P*=.02], GP [*F*(1, 177.72)=13.00, *P*<.001], EAP [*F*(1, 180.79)=4.20, *P*=.02], and phone helpline [*F*(1, 173.64)=3.18, *P*=.04].

**Table 1.4.** Observed means and standard deviations for individual help seeking intentions

|  | **Timepoint** | | | | | |
| --- | --- | --- | --- | --- | --- | --- |
|  | Pre-test | | Post-test | | 6-month follow-up | |
|  | *n* | *Mean (SD)* | *n* | *Mean (SD)* | *n* | *Mean (SD)* |
| *Help seeking intentions (Partner, friend parent, other relative/family member, etc.)* | | | | | | |
| Control | 263 | 6.09 (1.18) | 205 | 6.12 (1.20) | 54 | 6.46 (0.72) |
| Helipad | 223 | 6.17 (1.22) | 137 | 6.30 (1.13) | 51 | 6.29 (1.15) |
| *Help seeking intentions (psychologist)* | | | | | | |
| Control | 262 | 4.36 (1.83) | 202 | 4.62 (1.70) | 54 | 4.69 (1.56) |
| Helipad | 223 | 4.43 (1.80) | 140 | 5.00 (1.60) | 49 | 5.18 (1.35) |
| *Help seeking intentions (psychiatrist)* | | | | | | |
| Control | 262 | 3.01 (1.71) | 202 | 3.20 (1.73) | 54 | 3.04 (1.47) |
| Helipad | 220 | 2.79 (1.62) | 140 | **3.31 (1.60) ^a^** | 49 | 3.39 (1.67) |
| *Help seeking intentions (GP)* | | | | | | |
| Control | 262 | 4.31 (1.84) | 202 | 4.49 (1.85) | 54 | 4.54 (1.66) |
| Helipad | 222 | 4.12 (1.80) | 138 | **4.90 (1.65) ^b^** | 49 | 4.43 (1.71) |
| *Help seeking intentions (EAP)* | | | | | | |
| Control | 261 | 3.80 (1.73) | 203 | 4.01 (1.72) | 54 | 4.41 (1.58) |
| Helipad | 223 | 3.75 (1.86) | 139 | **4.30 (1.84) ^c^** | 50 | 4.52 (1.73) |
| *Help seeking intentions (Phone help line)* | | | | | | |
| Control | 262 | 2.88 (1.62) | 204 | 3.00 (1.66) | 54 | 2.96 (1.50) |
| Helipad | 223 | 2.70 (1.53) | 141 | **3.11 (1.68) ^d^** | 50 | 3.18 (1.47) |
| *Help seeking intentions (Internet program)* | | | | | | |
| Control | 260 | 3.14 (1.63) | 201 | 3.27 (1.68) | 54 | 3.50 (1.46) |
| Helipad | 223 | 2.96 (1.67) | 138 | **3.32 (1.74) ^e^** | 50 | 3.56 (1.68)  . |
| *Help seeking intentions (I would not seek help from anyone)* | | | | | | |
| Control | 261 | 2.56 (1.72) | 202 | 2.13 (1.50) | 54 | 2.56 (1.57) |
| Helipad | 219 | 2.69 (1.87) | 137 | 2.07 (1.61) | 50 | **2.20 (1.51) ^f^** |

Note: Significant changes were as follows: **^a^** pre-test to post-test vs. control, *t*(359.62)=-2.79, *P*=.01); **^b^** pre-test to post-test vs. control, *t*(361.59)=-5.00, *P*<.001; **^c^** pre-test to post-test vs. control, *t*(359.34)=-2.68, *P*=.01; **^d^** pre-test to post-test vs. control, *t*(353.99)=-2.48, *P*=.01; **^e^** pre-test to post-test vs. control, *t*(357.69)=-2.04, *P*=.04; **^f^** pre-test to 6-month follow-up vs. control, *t*(120.23)=2.02, *P*=.045.

### Partner, friend, parent, other relative/family member

Planned contrasts showed no significant changes over time for either condition.

### Psychologist

Planned contrasts showed that both conditions showed a significant increase in help seeking intentions - psychologist from pre-test to post-test [control *t*(368.10)=2.84, *P*=.01); Helipad [*t*(381.72)=4.84, *P*<.001)], but only the Helipad condition was significant from pre-test to 6-month follow-up [*t*(133.185)=3.64, *P<*.001), control *t*(131.25)=1.32, *P*=.19).

### Psychiatrist

Planned contrasts showed that both conditions showed a significant increase in help seeking intentions from a psychiatrist from pre-test to post-test [control *t*(352.81)=2.25, *P*=.03); Helipad [*t*(363.71)=5.53, *P*<.001)], but only the Helipad condition was significant from pre-test to 6-month follow-up [control, *t*(117.56)=1.21, *P*=.228), Helipad *t*(120.59)=2.23, *P=*.03)].

### GP

Only the Helipad condition showed a significant increase in help seeking intentions from a GP from pre-test to post-test as shown by planned contrasts [control *t*(356.86)=1.56, *P*=.12); Helipad *t*(364.05)=7.82, *P*<.001)], but the reverse was true from pre-test to 6-month follow-up, with only control condition significant [control *t*(113.79)=2.11, *P=*.04), Helipad *t*(114.78=1.76, *P*=.08).

### EAP

Again, planned contrasts showed that only the Helipad condition showed a significant increase in help seeking intentions from an EAP from pre-test to post-test [control *t*(354.49)=1.32, *P*=.19); Helipad *t*(361.88)=4.60, *P*<.001)]. However, both conditions showed a significant increase in help seeking intentions from an EAP from pre-test to 6-month follow up [control *t*(116.39)=2.91, *P=*.004), Helipad *t*(117.67)=2.19, *P*=.03).

### Phone helpline

The planned contrasts indicated only the Helipad condition showed a significant increase in help seeking intentions from a phone helpline from pre-test to post-test [control *t*(350.65)=1.29 *P*=.20); Helipad *t*(355.78)=4.31, *P*<.001)]. This pattern was similar at 6-month follow-up with only the Helipad condition showing a significant increase in help seeking intentions from an phone helpline from pre-test to 6-month follow up [control *t*(110.28)=1.24, *P=*.22), Helipad *t*(111.21)=2.36, *P*=.02).

### Internet program

Only the Helipad condition was shown via planned contrasts to display a significant increase in help seeking intentions from an internet program from pre-test to post-test [control *t*(353.09)=1.36, *P*=.18); Helipad *t*(360.07)=3.80, *P*<.001)]. However, both conditions showed a significant increase in help seeking intentions from an internet program from pre-test to 6-month follow up [control *t*(112.37)=2.22, *P=*.03), Helipad *t*(113.29)=3.01, *P*=.003).

### Would not seek help from anyone

Planned contrasts showed that both conditions showed a significant decrease (a positive finding in this case) in help seeking intentions to not seek help from anyone from pre-test to post-test [control *t*(368.68)=-4.48, *P*<.001); Helipad [*t*(386.39)=-5.00, *P*<.001)], but only the Helipad condition was significant from pre-test to 6-month follow-up [control, *t*(117.86)=0.46, *P*=.65), Helipad *t*(122.38)=-2.35, *P=*.02)].

## Help seeking behavior

Tables 1.5 presents participants meeting cut-off criteria on either PROMIS scale for each timepoint and help seeking behavior for each source on the AHSQ for this group with elevated symptoms (H3). Those meeting the cut-off (T-score ≥60) for either the Depression or Anxiety PROMIS measures at either pre-test or 6-month follow-up (*n*=179; 36.8%) were comparable across conditions Helipad (*n*=83; 37.2%) and control (*n*=96; 36.4%).

**Table 1.5.** Help seeking behavior from specific sources among participants with elevated symptoms of depression or anxiety at either post-test or 6-month follow-up (n=179)

|  | **Timepoint** | | | | | |
| --- | --- | --- | --- | --- | --- | --- |
|  | Pre-test | | Post-test | | 6-month follow-up | |
|  | *n* | *n (%)* | *n* | *n (%)* | *n* | *n (%)* |
| *Help seeking behavior (AHSQ), n (%) – Partner, friend, parent, other relative/family member* | | | | | | |
| Control | 96 | 43 (44.8) | -- | -- | 25 | 12 (48.0) |
| Helipad | 83 | 37 (44.6) | -- | -- | 27 | 12 (44.4) |
| *Help seeking behavior (AHSQ), n (%) – Psychologist* | | | | | | |
| Control | 96 | 36 (37.5) | -- | -- | 25 | 12 (48.0) |
| Helipad | 83 | 30 (36.1) | -- | -- | 27 | 8 (29.6) |
| *Help seeking behavior (AHSQ), n (%) – Psychiatrist* | | | | | | |
| Control | 96 | 7 (7.3) | -- | -- | 25 | 2 (8.0) |
| Helipad | 83 | 7 (8.4) | -- | -- | 27 | 2 (7.4) |
| *Help seeking behavior (AHSQ), n (%) – GP* | | | | | | |
| Control | 96 | 29 (30.2) | -- | -- | 25 | 9 (36.0) |
| Helipad | 83 | 25 (30.1) | -- | -- | 27 | 6 (22.0) |
| *Help seeking behavior (AHSQ), n (%) – EAP* | | | | | | |
| Control | 96 | 10 (10.4) | -- | -- | 25 | 5 (20.0) |
| Helipad | 83 | 11 (13.3) | -- | -- | 27 | 3 (11.1) |
| *Help seeking behavior (AHSQ), n (%) – Phone help line* | | | | | | |
| Control | 96 | 6 (6.3) | -- | -- | 25 | 0 (0.0) |
| Helipad | 83 | 3 (3.6) | -- | -- | 27 | 0 (0.0) |
| *Help seeking behavior (AHSQ), n (%) – Internet program* | | | | | | |
| Control | 96 | 2 (2.1) | -- | -- | 25 | 3 (12.0) |
| Helipad | 83 | 4 (4.8) | -- | -- | 27 | 1 (3.7) |

## *Note:* Participants who did not select either yes or no for the item on whether they had sought help or not and were not included in the data.

There was no significant interaction between condition and time (pre-test to 6-month follow-up) for help seeking behavior from a partner, friend, parent, other relative/family member (OR=0.86, 95% CI=0.24-3.04, *P*=.81), psychologist (OR=0.47, 95% CI =0.13-1.78, *P*=.27), psychiatrist (OR=0.78, 95% CI=0.07-8.25, *P*=.83), GP (OR=0.50, 95% CI=0.12-2.05 *P*=.34), EAP (OR=0.38, 95% CI=0.06-2.36, *P*= .29), Phone help line (OR=1.76, 95% CI=0.42-7.37, *P=*.44), or Internet (OR=0.12, 95% CI=0.01-2.26 *P*=.16.

Table 1.6 presents help seeking behavior for each source on the AHSQ for the full sample.

**Table 1.6.** Participants reporting help seeking behavior from individual sources (*n*=484).

|  | **Timepoint** | | | | | |
| --- | --- | --- | --- | --- | --- | --- |
|  | Pre-test | | Post-test | | 6-month follow-up | |
|  | *n* | *n (%)* | *n* | *n (%)* | *n* | *n (%)* |
| *Help seeking behavior (AHSQ), n (%) – Partner, friend, parent, other relative/family member* | | | | | | |
| Control | 263 | 77 (29.2) ( | -- | -- | 62 | 18 (29.0) |
| Helipad | 221 | 69 (30.9) | -- | -- | 59 | 19 (32.2) |
| *Help seeking behavior (AHSQ), n (%) – Psychologist* | | | | | | |
| Control | 263 | 59 (22.3) | -- | -- | 62 | 15 (24.2) |
| Helipad | 221 | 48 (21.5) | -- | -- | 59 | 12 (20.3) |
| *Help seeking behavior (AHSQ), n (%) – Psychiatrist* | | | | | | |
| Control | 263 | 11 (4.2) | -- | -- | 62 | 2 (3.2) |
| Helipad | 221 | 8 (3.6) | -- | -- | 59 | 2 (3.4) |
| *Help seeking behavior (AHSQ), n (%) – GP* | | | | | | |
| Control | 263 | 53 (20.1) | -- | -- | 62 | 13 (21.0) |
| Helipad | 221 | 41 (18.4) | -- | -- | 59 | 9 (15.3) |
| *Help seeking behavior (AHSQ), n (%) – EAP* | | | | | | |
| Control | 263 | 22 (8.3) | -- | -- | 62 | 8 (12.9) |
| Helipad | 221 | 19 (8.5) | -- | -- | 59 | 4 (6.8) |
| *Help seeking behavior (AHSQ), n (%) – Phone help line* | | | | | | |
| Control | 263 | 8 (3.0) | -- | -- | 62 | 0 (0) |
| Helipad | 221 | 3 (1.3) | -- | -- | 59 | 0 (0) |
| *Help seeking behavior (AHSQ), n (%) – Internet program* | | | | | | |
| Control | 263 | 3 (1.1) | -- | -- | 62 | 4 (6.5) |
| Helipad | 221 | 6 (2.7) | -- | -- | 59 | 2 (3.4) |

***Note:*** Participants who did not select either yes or no for the item on whether they had sought help or not and were not included in the data.

There was no significant interaction between condition and time (pre-test to 6-month follow-up) for help seeking behavior from a partner, friend, parent, other relative/family member (OR=1.06, 95% CI=0.44-2.54, *P=*.90), psychologist (OR=0.83, 95% CI=0.32-2.20, *P=*.71), psychiatrist (OR=1.22, 95% CI=0.13-11.26, *P=*.86), GP (OR=0.75, 95% CI=0.26-2.15, *P=*.59), EAP (OR=0.48, 95% CI=0.12-1.98, *P=*.31), Phone help line (OR=2.28, 95% CI=0.59-8.75, *P=*.23), or Internet (OR=0.21, 95% CI=0.02-1.99 *P=*.17).

### Moderators of intentions

Figure 1.1 demonstrates the effect of pre-test distress on help seeking intentions by condition using a binary model of distress on the DQ5 (0-10=low distress; 11-25=high distress). This model was also significant, (*F*(2,179.48)=3.99, *P*=.02). The figure shows that in the Helipad condition improved intentions over time whether their distress was high or low at pre-test; however, for the control group, only those with high distress at pre-test improved intentions over time. Those with low distress showed little or no change over time.

Figure 1.1. Estimated marginal means and standard errors for help seeking intentions from professional sources (GHSQ) for each condition over time and by distress level (binary).

**Box 1.2.** Helipad 6-month reminder email sent to employees

**Subject line: *HeLiPaD program 6-month follow up***

Hi there,

We would like to invite you to please complete a brief survey **(10 minutes)** to check up on how you’re doing after participating in our study.

Please CLICK HERE to access your survey.

**Thank you for your participation.**

Best wishes,

The HeLiPaD project team

The ethical aspects of this research have been approved by the ANU Human Research Ethics Committee (2023/053).
